# Cholinergic Activation of M2 Receptors Leads to Context-Dependent Modulation of Feedforward Inhibition in the Visual Thalamus

**DOI:** 10.1371/journal.pbio.1000348

**Published:** 2010-04-06

**Authors:** Miklos Antal, Claudio Acuna-Goycolea, R. Todd Pressler, Dawn M. Blitz, Wade G. Regehr

**Affiliations:** Department of Neurobiology, Harvard Medical School, Boston, Massachusetts, United States of America; European Brain Research Institute, Italy

## Abstract

In many brain regions, inhibition is mediated by numerous classes of specialized interneurons, but within the rodent dorsal lateral geniculate nucleus (dLGN), a single class of interneuron is present. dLGN interneurons inhibit thalamocortical (TC) neurons and regulate the activity of TC neurons evoked by retinal ganglion cells (RGCs), thereby controlling the visually evoked signals reaching the cortex. It is not known whether neuromodulation can regulate interneuron firing mode and the resulting inhibition. Here, we examine this in brain slices. We find that cholinergic modulation regulates the output mode of these interneurons and controls the resulting inhibition in a manner that is dependent on the level of afferent activity. When few RGCs are activated, acetylcholine suppresses synaptically evoked interneuron spiking, and strongly reduces disynaptic inhibition. In contrast, when many RGCs are coincidently activated, single stimuli promote the generation of a calcium spike, and stimulation with a brief train evokes prolonged plateau potentials lasting for many seconds that in turn lead to sustained inhibition. These findings indicate that cholinergic modulation regulates feedforward inhibition in a context-dependent manner.

## Abstract 2

The temporal dynamics of inhibition within a neural network is a crucial determinant of information processing. Here, the authors describe in the visual thalamus how neuromodulation governs the magnitude and time course of inhibition in an input-dependent way.

## Author Summary

Within the visual thalamus, a single type of inhibitory interneuron regulates activity evoked by retinal ganglion cells and controls the visual signals that reach the cortex. Here, we find that neuromodulation, of the sort thought to occur when an animal is attending to a task, regulates the firing mode of these interneurons and controls the resulting inhibition in an input-dependent manner. When few ganglion cells are activated, neuromodulation greatly decreases the number of spikes in interneurons, and as a result, strongly reduces the inhibition of relay neurons. This favors the lossless transmission of weak visual signals to the cortex by virtually eliminating inhibition within the thalamus. In contrast, when many ganglion cells are activated, the same neuromodulator leads to strong and prolonged inhibition. This is accomplished by promoting the generation of calcium spikes and prolonged depolarizations in interneurons. In this way, a modulator can regulate the flow of visual information in a context-dependent manner.

## Introduction

Local inhibitory interneurons are important circuit elements that can generate synchrony within populations of neurons, control synaptic integration, and regulate the spiking activity of principal cells. In many brain regions, inhibition is mediated by numerous types of interneurons that contact different domains of the principal cells and provide inhibition with distinct spatial and temporal properties [1]–[10]. Therefore, particular aspects of inhibition can be precisely regulated by activating different subsets of interneurons [11]–[14].

In other brain regions, such as the dorsal lateral geniculate nucleus (dLGN) of the thalamus, there is less diversity in the types of interneurons. In the rodent dLGN, there appears to be just one type of interneuron [15]–[17]. The dLGN is a vital link in the visual pathway from the eye to the visual cortex [16],[18]. Retinal ganglion cells (RGCs) activate thalamocortical (TC) neurons that convey visually evoked signals to the cortex, and local interneurons within the dLGN that provide feedforward inhibition to TC cells. dLGN interneurons control the number of visually evoked spikes, regulate the precision of firing, and refine the receptive fields (RFs) of TC neurons [19]–[24].

Even though there are not diverse interneuron subtypes within the dLGN, the distinctive properties of dLGN interneurons raise the possibility that neuromodulators might influence specific aspects of feedforward inhibition. dLGN interneurons release GABA from both their axons and dendrites [16],[25]. Their dendrites support both brief sodium action potentials and long-lasting calcium spikes, whereas their axons support only sodium spikes. Thus, active responses in the postsynaptic cell control the magnitude, spatial properties and time course of GABA release. The response mode of dLGN interneurons to RGC activation is highly sensitive to their membrane potential: depolarized interneurons tend to fire a single, short-latency sodium action potential, whereas hyperpolarization promotes calcium spikes and bursts of sodium spikes [15],[26]. It is not known whether neuromodulators can shift the response mode of dLGN interneurons and thereby affect the properties of inhibition onto TC neurons.

Here, we test the possibility that activation of dendritic muscarinic acetylcholine (ACh) receptors can regulate the properties of feedforward inhibition onto TC neurons by switching the firing mode of dLGN interneurons. Attention and arousal are thought to increase cholinergic tone, and cholinergic modulation mediated by muscarinic receptors is important in many brain regions [27]–[30]. Previous studies have established that cholinergic inputs provide an important modulatory signal to cells in the dLGN [31]. Interneurons within the dLGN express muscarinic type-2 (M2) receptors [32]. Activation of these receptors is known to open potassium channels, hyperpolarize dLGN interneurons [33],[34], and decrease the spontaneous inhibitory input onto TC neurons [35]. We find that activation of M2 receptors regulates feedforward inhibition following activation of RGC synapses in a manner that is dependent on the number of RGCs activated. When moderate stimulus intensities are used to activate RGC axons with a single stimulus, ACh makes it more difficult to evoke an action potential in dLGN interneurons. As a result, when a modest number of RGC axons are activated, ACh virtually eliminates feedforward inhibition. In contrast, when many RGC axons are activated with a single stimulus, muscarine promotes the generation of calcium spikes and bursts of sodium spikes in interneurons and leads to long-lasting inhibition with little change in the overall inhibitory charge. Remarkably, in the presence of muscarine, the synchronous activation of many RGCs with brief stimulus trains (five at 10 Hz) produces a long-lasting plateau potential in interneurons and a sustained component of inhibition in TC neurons that last for tens of seconds. Thus, ACh is able to control the magnitude and time course of feedforward inhibition in a context-dependent manner.

## Results

### Responses of dLGN Interneurons to Optic Tract Stimulation

**Figure 1:**
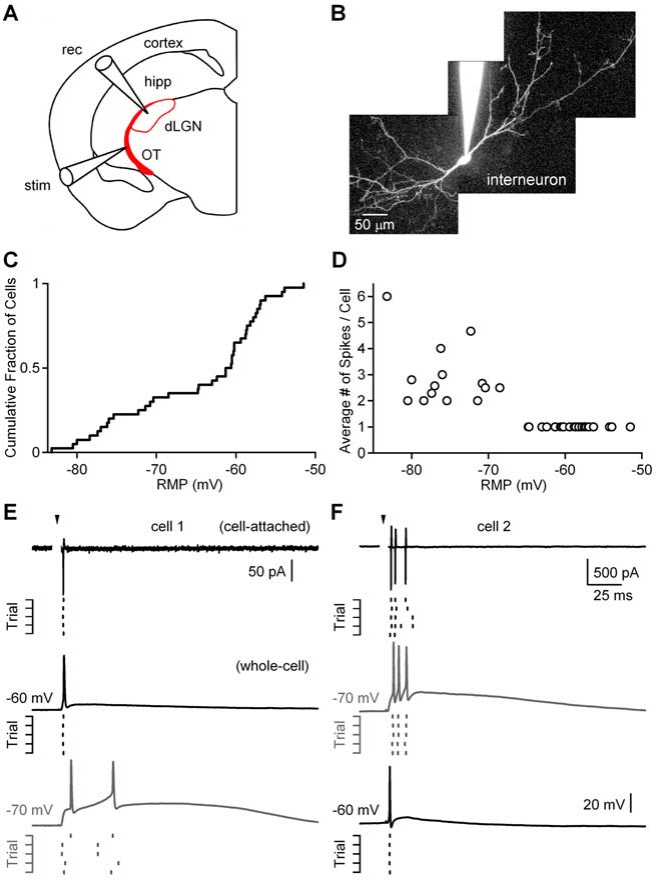
Synaptically evoked responses in dLGN interneurons are dependent on resting membrane potential. (A) Experimental configuration is shown for a pseudosaggital brain slice with a stimulus electrode (stim) placed in the optic tract (OT) and a recording electrode (rec) within the dLGN. hipp, hippocampus. (B) A two-photon fluorescence image of a dLGN interneuron is shown with Alexa-594 (50 µM) in the recording pipette. (C and D) Measurements of resting membrane potential (RMP) and action potentials were made from dLGN interneurons with cell-attached recordings with potassium-filled electrodes (see Figure S1). (C) A cumulative histogram of the RMP of interneurons (*n* = 40 cells). (D) The number of spikes evoked in the 250 ms following strong OT stimulation is plotted as a function of the RMP for each cell. (E and F) Representative recordings reveal two types of interneuron responses to OT stimulation. In some cases, OT stimulation evoked a single, short-latency spike that could be detected in cell-attached mode, as shown for a representative trace and a raster plot ([E], top). After breaking into the cell, a single sodium action potential was reliably evoked ([E], middle). When the cell was hyperpolarized by injecting negative current, the same stimulus evoked a calcium spike and two sodium spikes ([E], bottom gray). In another experiment (F), OT stimulation evoked a burst of spikes as detected with an on-cell electrode ([F], top). After obtaining a whole-cell recording, it was found that the sodium spikes were riding on a calcium spike ([F], middle gray). When the dLGN interneuron was depolarized with positive current injection, OT stimulation evoked a single sodium action potential ([F], bottom). Stimulus artifacts were digitally removed for clarity. Arrowheads indicate timing of stimulation. Time scale bar in (F) also applies to (E).

An important characteristic of interneurons within the dLGN is that they can fire in different modes following optic tract (OT) stimulation, and resting membrane potential can be an important factor in controlling the firing mode [15],[26]. Therefore, before examining the regulation of disynaptic inhibition, we used a noninvasive means to measure the resting potential of dLGN interneurons in control conditions and determine how this influences the responses evoked by strong OT stimulation (see Materials and Methods). Axons from RGCs were activated with a stimulus electrode placed in the OT, and responses were monitored in the dLGN in pseudosagittal brain slices (Figure 1A and 1B). In these experiments, we obtained cell-attached recordings with pipettes containing a high concentration of potassium. Potassium currents measured in response to a voltage ramp allowed us to determine the resting potentials (Figure S1, [36]–[38]). We found that there was an extremely broad range of resting potentials (Figure 1C, median, interquartile range [IQR]: −61 mV, 14 mV, *n* = 40). We used the same electrodes to measure the sodium action potentials evoked by strong OT stimulation. The number of action potentials evoked by such stimulation depended upon the membrane potential of the interneuron (Figure 1D). Single action potentials were evoked in interneurons more depolarized than −66 mV, and multiple spikes were evoked for cells more negative than −66 mV. This raises the possibility that there are two different types of interneurons, one that tends to fire single spikes and the other that tends to fire in bursts. Another possibility is that these distinct responses represent distinct states of the postsynaptic cell that control the firing pattern evoked by OT stimulation.

To distinguish between these two possibilities, we performed experiments in which pipettes containing standard intracellular solution was used, and after recording responses in cell-attached configuration, a whole-cell recording was obtained from the same cell. OT stimulation evoked very similar responses in cell-attached and whole-cell modes. As shown in a representative experiment, a cell with a single, short-latency spike had a resting potential of −60 mV (Figure 1E, middle trace). Small current injections to hyperpolarize the cell to −70 mV changed the response to a pair of sodium spikes riding on a depolarizing afterpotential (Figure 1E, bottom gray trace). Previously, we have shown that this slow active response is a calcium spike that is mediated, at least in part, by L-type calcium channels [15]. In a different cell, OT stimulation evoked a burst of sodium spikes in both the cell-attached and whole-cell configurations (resting potential near −70 mV) (Figure 1F). In this case, when the cell was depolarized to −60 mV, the same OT stimulation evoked a single, short-latency spike (Figure 1F, bottom trace). These experiments suggest that dLGN interneurons are capable of responding with either a single spike or a burst, and that the initial resting membrane potential is an important determinant of the response of a given interneuron.

### Acetylcholine Differentially Modulates Synaptically Evoked Interneuron Responses

**Figure 2 pbio-1000348-g002:**
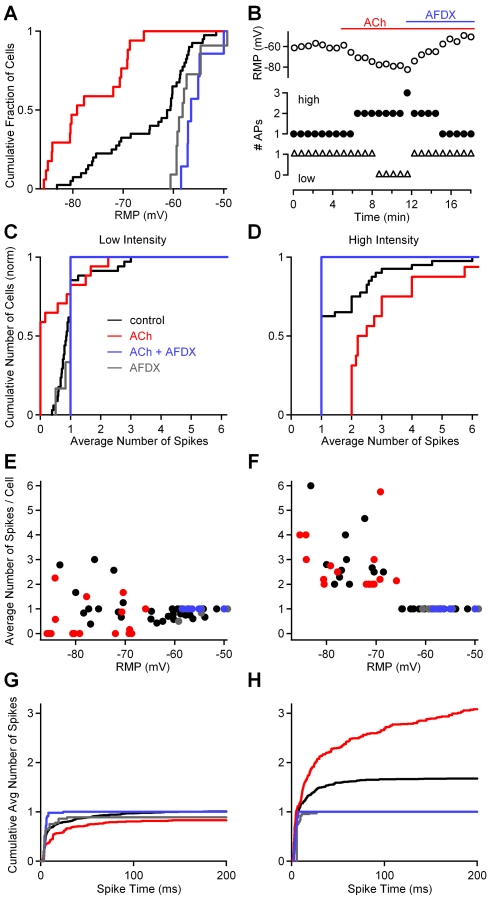
Acetylcholine modulates the responses of dLGN interneurons in an intensity-dependent manner. (A) Cumulative histogram showing the distribution of dLGN interneuron resting membrane potentials (RMPs) measured in a cell-attached configuration in control conditions (black), in the presence of acetylcholine (ACh) (red), in the presence of M2 receptor antagonist AFDX and ACh (blue) and in the presence of AFDX alone (gray). (B) A representative experiment is shown where the RMP and responses of a dLGN interneuron to OT stimulation were monitored. AP, action potential. (C–H) Experiments were performed in which either low- (C, E, and G) or high- (D, F, and H) intensity OT stimulation were used to activate dLGN interneurons. The responses evoked in different experimental conditions are summarized with cumulative histograms (C and D), the number of spikes evoked in each cell as a function of membrane potential (E and F), and the average number and timing of spikes evoked per trial (G and H).

Based on the observation that cholinergic activation can hyperpolarize dLGN interneurons [33],[34], we examined whether ACh could affect their firing mode following OT stimulation. We initially examined how ACh influenced the resting potential and found that bath application of ACh (100 µM) hyperpolarized dLGN interneurons by 18 mV (Figure 2A, median, IQR: −79 mV, 14 mV, *n* = 17 in ACh, *p*<0.0001 Kruskal-Wallis [K-W] ANOVA). Conversely, the application of the selective M2 receptor antagonist AF-DX116 (AFDX, 10 µM) in the continued presence of ACh led to a narrow range of resting potentials that were depolarized relative to control (Figure 2A, median, IQR: −57 mV, 2 mV, *n* = 7; *p* = 0.13, K-W ANOVA). These findings indicate that in control conditions, endogenous levels of ACh hyperpolarize dLGN interneurons by activating M2 receptors. Resting potentials during coapplication of ACh and AFDX were similar to those observed in AFDX alone (Figure 2A, median, IQR: −58 mV, 4 mV, *n* = 11, *p* = 0.53, K-W ANOVA), indicating that ACh controls the resting potential of dLGN interneurons primarily by activating M2 receptors.

To determine whether this M2 receptor–mediated hyperpolarization has an influence on interneuron output, single electrical stimuli were delivered to the OT at low and high intensities and spiking responses were monitored in cell-attached recordings. A representative experiment is shown in which the resting potential and the responses of a dLGN interneuron to OT stimulation were monitored before and after cholinergic activation (Figure 2B). In control conditions, the cell fired a single spike at both low and high stimulus intensities. Bath application of ACh hyperpolarized the cell, which no longer fired single spikes but, depending on the intensity of the stimulus, was either unable to fire at low stimulus intensities or fired bursts when stimulating at high intensities. These effects were reversed in the presence of the M2 antagonist. Thus, in a single dLGN interneuron, the response to OT stimulation depends upon the number of RGCs that are activated.

The responses of dLGN interneurons to a single, low-intensity OT stimulation (∼25 µA) are summarized (Figure 2C, 2E, and 2G). In control conditions, 62% of the cells either fired a single, short-latency spike or failed to fire, with the average number of spikes per trial between 0 and 1. Twenty-three percent of cells always fired once, and 15% fired multiple, longer-latency spikes. In the presence of ACh, OT stimulation was no longer able to elicit active response in 59% of the cells; in 18% of cells, the average number of spikes per trial was between 0 and 1, 6% of cells always fired one spike; and 17% fired a burst of sodium spikes (Figure 2C, 2E, and 2G). At low stimulus intensities, the probability of evoking an active response was significantly reduced by ACh application (*p* = 0.004, K-W ANOVA). M2 receptor blockade reversed this effect, as indicated by the average number of spikes per trial for a given cell, which was one in all recordings (*n* = 7, *p* = 0.0008, K-W ANOVA). The responses of dLGN interneurons were similar in the presence of the M2 antagonist plus ACh, and in the presence of the M2 antagonist alone: 33% of six cells fired one spike or were unable to fire, and 67% of cells always fired a single spike (*p* = 0.38, K-W ANOVA). Thus, at low-intensity stimulation, ACh acts to suppress interneuron firing.

The effect of ACh on the responses of dLGN interneurons evoked by single, high-intensity OT stimulation (between 100 and 200 µA, see Materials and Methods) were very different (Figure 2D, 2F, and 2H) from those evoked by low-intensity stimulation. In control conditions, 65% of interneurons always fired a short-latency single spike, and 35% fired a burst of sodium spikes. In the presence of ACh, all cells fired bursts of sodium spikes, and the number of spikes was larger in the presence of ACh relative to control conditions (*n* = 16, *p*<0.0001, K-W ANOVA). In the presence of the M2 antagonist alone and in the presence of both ACh and the antagonist, strong OT stimulation always evoked a single, short-latency spike (Figure 2D, 2F, and 2H), and there was a significant decrease in the number of evoked spikes compared to control (*n* = 11, *p* = 0.01; and *n* = 7, *p* = 0.03, respectively, K-W ANOVA). The cumulative histogram of the number of action potentials evoked per trial reveals that for low-intensity stimulation, ACh decreased the number of evoked sodium spikes (Figure 2G), whereas for high-intensity stimulation, ACh increased the number of sodium spikes by increasing the number of longer-latency sodium spikes (Figure 2H). ACh increased the median and IQR of spike times from 4 ms and 12 ms to 13 ms and 43 ms, respectively (*p*<0.0001, K-W ANOVA). Hence, in contrast to stimulation at low intensities, ACh promotes burst firing in response to single, high-intensity stimuli in dLGN interneurons.

These cell-attached recordings indicate that there exists a basal cholinergic tone under our experimental conditions that strongly influences the way dLGN interneurons respond to sensory input by activating M2 receptors. Previous studies suggest that basal ACh levels, which have been estimated to be in the high nanomolar to low micromolar range [39]–[41], can regulate neuronal activity in other brain regions [42]. This can occur by activation of nicotinic ACh receptors [43],[44], muscarinic M1 receptors [45], M3 receptors [46], and M1-M4 receptors [47]. Among classical neurotransmitters, ACh is the only one to be removed, not by reuptake, but through degradation by ACh-esterase (AChE) [40], and regulation of esterase activity would be a viable means of regulating the ambient levels of ACh and the state of LGN interneurons.

**Figure 3:**
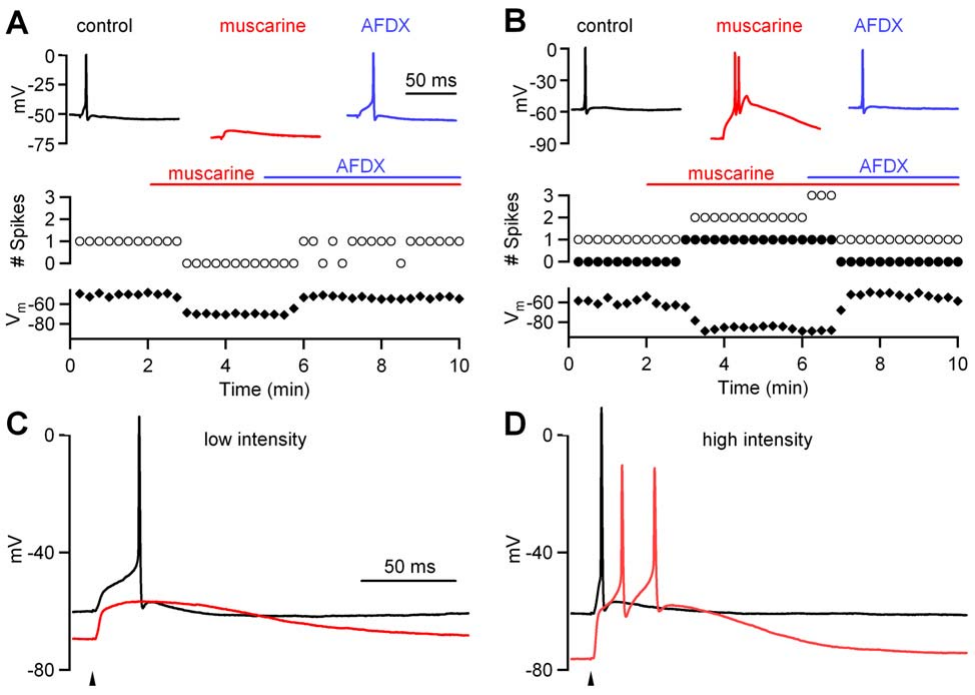
Differential effects of muscarine on interneuron responses evoked by synaptic stimulation. (A) A representative experiment is shown in which interneuron responses were evoked by low-intensity OT stimulation just above the threshold to initiate an action potential. In control conditions (black trace), a single sodium spike was triggered, whereas in the presence of muscarine (red trace), the neuron was hyperpolarized and no spikes were evoked. In the presence of the M2 receptor antagonist AFDX, the cell returned to its initial membrane potential (blue trace), and a single spike was again evoked. The number of sodium spikes (open circles) and the membrane potential (black diamonds) are plotted as a function of time. (B) In another representative experiment, strong OT stimulation evoked a single sodium spike in control conditions, whereas in the presence of muscarine, the same stimulus evoked a calcium spike and multiple sodium spikes. The number of sodium spikes (open circles), calcium spikes (closed circles), and the membrane potential (black diamonds) are plotted as a function of time. Time scale bar in (A) also applies to (B). (C and D) Responses of the same dLGN interneuron to OT stimulation with low (C) and high (D) intensities are shown for control conditions (black traces) and in the presence of muscarine (red traces). Arrowheads indicate timing of stimulation. Time scale bar in (C) also applies to (D).

We also used whole-cell recordings to examine the effects of M2 receptor activation on dLGN interneuron responses (Figure 3). In these experiments, we used muscarine (2 µM), and the M2 receptor antagonist AFDX (10 µM). We focused on cells that initially had relatively depolarized membrane potentials and therefore responded to OT stimulation with a single, short-latency sodium spike. We evoked responses with OT stimulation that was slightly above threshold for evoking a spike in control conditions (low stimulation intensity, Figure 3A). In the presence of muscarine, the same-intensity OT stimulation was unable to evoke a spike. Muscarine actions on both interneuron membrane potential and spike probability were reversed by application of the M2 receptor antagonist. For all experiments (*n* = 5) in which low-intensity OT stimulation reliably evoked a single sodium spike in control conditions, the same stimulus was unable to trigger a spike in the presence of muscarine.

When the OT was activated with large amplitude stimulation (near 200 µA), muscarine had a very different effect on interneuron firing. As shown for a representative recording (Figure 3B), OT stimulation that triggered a single sodium spike in control conditions increased the number of evoked sodium spikes (from one to three in this example) and reliably evoked a calcium spike in the presence of muscarine. These effects of muscarine on firing were also accompanied by hyperpolarization of the cell. Similar results were seen in five experiments in which the OT was activated with high-intensity stimulation, and the cells responded with a single, short-latency action potential in control conditions. These experiments suggest that the actions of muscarine on interneuron firing depend on the number of convergent retinal ganglion cells activated by OT stimulation.

We then directly examined the stimulus dependence of modulation by studying the relationship between OT stimulus intensity and muscarinic modulation in the same, individual dLGN interneurons (Figure 3C and 3D). This is shown in a representative experiment in which the OT was stimulated with an intensity adjusted to just above the threshold to evoke a single, long-latency spike. Higher stimulus intensities also evoked single, short-latency spikes (unpublished data). In the presence of muscarine, the cell was hyperpolarized from a resting potential of −60 mV in control conditions to −75 mV. Low stimulus intensities failed to evoke a sodium spike in muscarine. Higher stimulus intensities evoked two sodium spikes riding on a calcium spike. A comparison of responses evoked in dLGN interneurons at low and high intensities in control conditions and in the presence of muscarine illustrates how muscarinic modulation depends on the stimulus conditions (Figure 3C and 3D). Similar results were observed in 29 additional experiments. Because activation of muscarinic receptors does not affect transmitter release from RGC axons onto interneurons (Figure S2), the responses measured here reflect activation of receptors on dLGN interneurons. Thus, consistent with our previous findings, the actions of muscarine on dLGN interneuron firing are also intensity dependent. At low OT stimulation intensities, muscarine virtually eliminates interneuron firing. In contrast, at higher stimulation intensities, muscarine can switch the firing mode of dLGN interneurons from a single sodium action potential to a burst of sodium and calcium spikes.

Anatomical studies in cats suggest that relay neurons may make collaterals onto interneurons [48]. We therefore considered the possibility that increasing the intensity of OT stimulation could promote disynaptic inhibition as a result of relay neurons more effectively recruiting interneurons. If such recurrent excitation were prominent in mice, then there would need to be both monosynaptic excitatory postsynaptic currents (EPSCs) of OT inputs onto dLGN interneurons, followed by later disynaptic EPSCs from relay neurons. We never observed any such disynaptic excitation (unpublished data), indicating that under our experimental conditions, recurrent excitation of dLGN interneurons is absent and therefore does not contribute to the disynaptic inhibition we observe in TC neurons.

We also performed experiments under conditions in which we did not perturb the interneurons with a whole-cell recording while GABA_A_ and GABA_B_ signaling was intact (Figure S3A–3C). In these experiments, we measured on-cell responses evoked by high-intensity stimulation in control conditions, in the presence of muscarine, and following its washout (Figure S3A). With inhibition intact, 82% (23/28) of cells fired a single spike per trial in control conditions, and the remaining cells (18%) fired multiple spikes per trial (Figure S3B, black trace). In the presence of muscarine, fewer cells fired a single spike (43%), whereas 54% fired multiple spikes per trial, and 3% showed no response (Figure S3B, red trace). A plot of the cumulative number of spikes per trial for the 28 cells revealed that in control conditions, and following the washout of muscarine, most spikes occurred several milliseconds after OT stimulation, whereas in the presence of muscarine, many of the spikes occurred tens of milliseconds after OT stimulation (Figure S3C). The findings are consistent with OT stimulation having a greater tendency to elicit calcium spikes in dLGN interneurons in the presence of muscarine even with synaptic inhibition intact.

The effect of muscarine on the response of dLGN interneurons to strong OT stimulation suggests a rather unconventional mechanism of modulation in which muscarine could promote the initiation of a dendritic calcium spike in response to OT stimulation. It has previously been shown that calcium spikes in dLGN interneurons are mediated in part by NMDA receptors and L-type calcium channels and that they lead to large dendritic calcium transients and promote long-latency disynaptic inhibition [15]. We tested the idea that muscarine greatly increases the dendritic calcium signals evoked by strong OT stimulation by using two-photon calcium imaging (Figure S4). High-intensity OT stimulation that triggered a single sodium spike in interneurons evoked fast calcium signals in their dendrites. Muscarine hyperpolarized the cells, switched their firing mode from a single sodium spike to a burst of sodium spikes and a calcium spike, and evoked larger and longer-lasting dendritic calcium elevations (4-fold increase, *n* = 3). These findings show that, by switching the mode of interneuron firing, muscarine can promote larger calcium signals that could trigger GABA release from dLGN interneuron dendrites.

### Context-Dependent Actions of Muscarine on Local Feedforward Inhibition

Previous studies have suggested that, by opening potassium channels and both hyperpolarizing the cell and lowering its input resistance, muscarine can make it more difficult to trigger sodium spikes in dLGN interneurons [33],[34]. As a result, moderate-intensity OT stimulation is typically insufficient to trigger a spike in the presence of muscarine. We show here that muscarine also increases the tendency of neurons to fire calcium spikes in response to OT stimulation, provided the stimulus is sufficiently strong. As a result, by promoting calcium spikes and late sodium spikes, muscarine evoked more prolonged calcium elevations and has the potential to increase long-latency GABA release provided by dLGN interneurons onto TC neurons.

**Figure 4:**
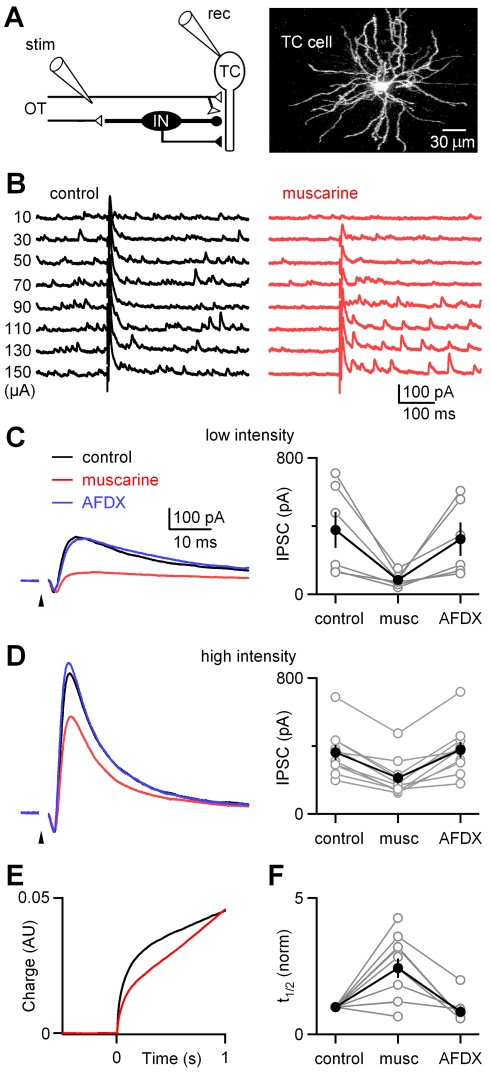
Context-dependent muscarinic modulation of feedforward inhibition within the dLGN. (A) Left: schematic of experimental configuration. The OT was stimulated (stim) by an extracellular electrode, and disynaptic inhibitory currents were recorded (rec) from TC cells. Right: 2-photon fluorescence image) in voltage-clamp at a holding potential of −10 mV. Disynaptic inhibitory currents in TC neurons are the result of RGC axons activating dLGN interneurons (IN), which in turn release GABA from their dendrites and axons. Under these conditions, the inhibitory and excitatory currents were outward and inward, respectively. The EPSC was also monitored and was found to be unaffected by muscarine. (B) In a representative experiment, a range of stimulus intensities was used to activate the OT, and responses were measured in control conditions (left) and in the presence of muscarine (right). (C and D) Representative experiments and summaries are shown on an expanded time scale for OT stimulation of low-intensity (C) and high-intensity (D) stimulation. Stimulus artifacts were digitally removed for clarity. Arrowheads indicate timing of stimuli. musc, muscarine. (E) The time course of inhibition evoked by high-intensity stimulation was quantified by integrating the inhibitory current. The average synaptic charge is shown for experiments (*n* = 10) with high-intensity stimulation. AU, arbitrary units. (F) The time at which the synaptic currents reached half of the amplitude of the total synaptic charge 1 s after stimulating the OT was determined, and the half-times were normalized relative to those measured in control conditions. Individual experiments (open circles) and average half-times (closed circles) are plotted.

We tested this idea by stimulating the OT at progressively higher intensities while recording feedforward inhibitory currents in TC cells before and after muscarine application. Under our experimental conditions, disynaptic inhibitory currents recorded in TC neurons are the result of RGC axons activating dLGN interneurons, which in turn release GABA from their dendrites and axons (Figure 4A). The muscarinic actions on disynaptic inhibition were exclusively due to activation of M2 receptors in interneuron dendrites (Figure S5). As the stimulus intensity was increased in control conditions, a progressively larger rapid inhibitory component was evoked (Figure 4B, left). Muscarine greatly enhanced the long-latency inhibitory events that follow high-intensity OT stimulation (Figure 4B, right). In this representative example, muscarine reduced the rapid component of inhibition evoked by 30-µA stimulation to about 30% of control levels, but the rapid component was reduced only to about 70% of control levels for 150 µA. Muscarine also reduced the frequency of spontaneous inhibitory currents recorded in TC cells, as described previously [25]. Thus, the intensity dependence of suppression found here is consistent with the observed dependence of the dLGN interneuron responses on the intensity of OT stimulation.

In order to quantify the dependence of muscarinic modulation on the intensity of OT stimulation, we performed a series of experiments in which TC neurons were stimulated at a constant intensity in control conditions, in the presence of muscarine, and in the presence of AFDX. Representative traces are shown for low-intensity stimulation (Figure 4C, left) and high-intensity stimulation (Figure 4D, left). Again, muscarine had a much more pronounced effect on the rapid component of inhibition for low-intensity stimulation (Figure 4C, right, reduced to 29±6% [mean ± standard error of the mean (SEM)] of control, *n* = 6) than for high-intensity stimulation (Figure 4D, right, reduced to 58±4% [mean ± SEM] of control, *n* = 10). The differences in the extent of suppression for low- and high-intensity stimulus intensities were statistically significant (*p* = 0.001, Student paired *t*-test).

To determine whether muscarine affected the time course of inhibitory input onto TC neurons, the synaptic charge was calculated by integrating the evoked synaptic currents, and the time taken to reach half of the total synaptic charge was determined for each experiment (Figure 4E and 4F). In control conditions, there was considerable variability in the half-times, which ranged from 20 ms to 150 ms. Muscarine significantly increased the half-time (mean ± standard error of the mean [SEM]: 2.43±0.35-fold increase, *n* = 10, *p* = 0.006, Student *t*-test). During the subsequent application of AFDX, the half-times were similar to those in control conditions (the ratio of half-times in control and AFDX was 0.84±0.20 [mean ± SEM], *n* = 7, Student *t*-test, Figure 4F).

The prolongation of the time course of inhibition in the presence of muscarine influences the overall inhibition. This was justified by considering the synaptic charge within the first second after high-intensity stimulation of the OT (Figure 4E). Overall disynaptic inhibitory charge was not significantly altered (mean ± SEM: 1.00±0.16, *n* = 10, *p* = 0.93, Student *t*-test), thus, although there was a reduction in the early component of inhibition, muscarine shifted inhibition later in time without reducing its overall amount. During the subsequent application of AFDX, the integrated synaptic charge was not significantly different from control (mean ± SEM: 0.83±0.13, *n* = 7, *p* = 0.13, Student *t*-test).

### Optic Tract Activation with Brief Trains

**Figure 5:**
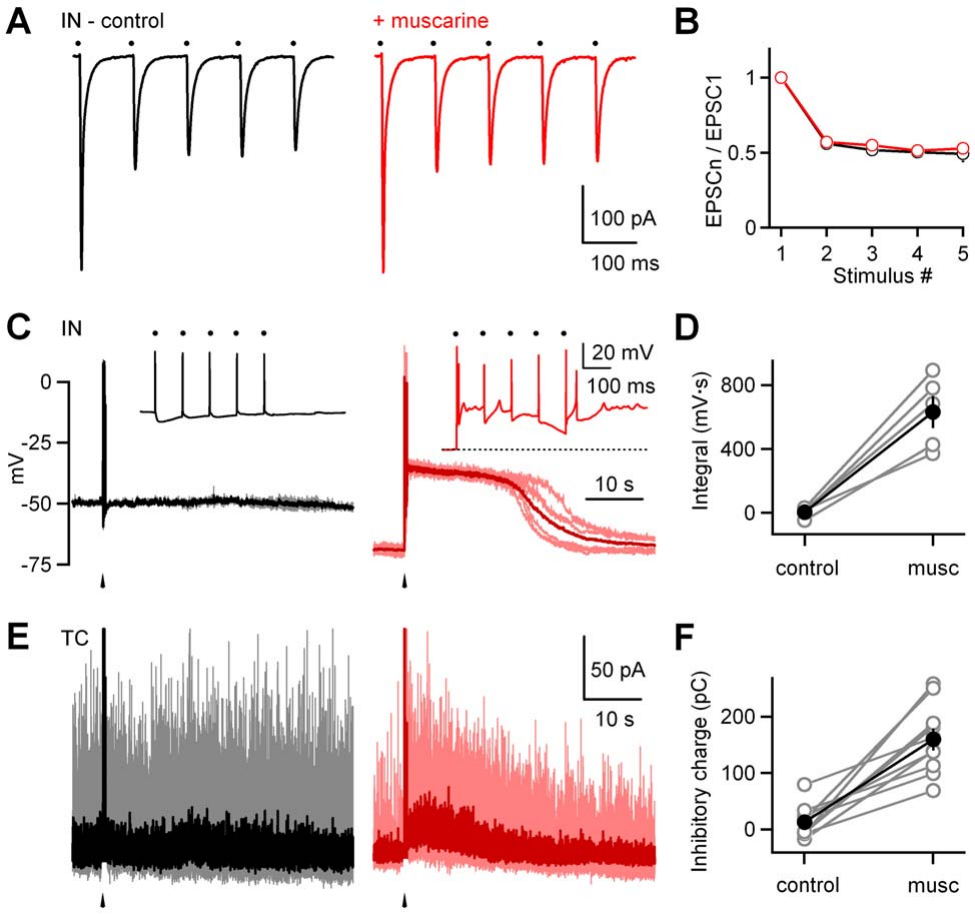
Muscarine promotes persistent activation of dLGN interneurons and long-lasting inhibition of TC neurons. Experiments were conducted in which the OT was activated with a train of five stimuli at 10 Hz at moderate intensity, and responses were measured in the absence and in the presence of muscarine (musc, 2 µM) in dLGN interneurons (IN) in voltage clamp (A and B) and in current clamp (C and D), and in TC neurons in voltage clamp (E and F). All experiments were conducted with GABA signaling intact. Representative recordings are shown in (A, C, and E), and are summarized in ([B], *n* = 4), ([D], *n* = 5), and ([F], *n* = 10), respectively. Dots in (A) and (C) indicate the timing of individual stimuli, and arrowheads in (C) and (E) indicate the timing of stimulus trains. Insets in (C) show responses on a finer time scale. In (C) and (E), consecutive traces (gray, light red) and their corresponding averages (black, dark red) are shown superimposed. Stimulus artifacts were digitally removed for clarity.

Initially, we focused on the effects of muscarine on responses evoked by single stimuli. We therefore extended these studies to more physiologically relevant stimuli by examining the effect of muscarine on responses evoked by repetitive activation of retinal ganglions cell axons (five stimuli of the OT at 10 Hz). Interneuron recordings showed that EPSC amplitudes exhibited short-term depression with repeated stimulation (Figure 5A, left). Muscarine did not affect the amplitude of the initial EPSC evoked in interneurons or short-term depression, as shown in a representative experiment (Figure 5A). The ratio of responses measured in muscarine and in control conditions (Figure 5B) illustrate muscarine did not affect either the amplitude (mean ± SEM: 0.99±0.04 fraction of control, *n* = 4, *p* = 0.7, Student *t*-test) or the paired-pulse ratio (mean ± SEM: 1.006±0.006, *n* = 4, *p* = 0.3, Student *t*-test) or the EPSC_5_/EPSC_1_ ratio (mean ± SEM: 1.09±0.05 fraction of control, *n* = 4, *p* = 0.13, Student *t*-test). As shown in a current-clamp recording, each OT stimulation evoked a sodium action potential in control conditions (Figure 5C, left). Remarkably, in the presence of muscarine, the same stimulation also evoked a long-lasting plateau potential that lasted for tens of seconds (Figure 5C, right). Sodium action potentials were only evoked during and shortly after the stimulus train, and did not persist throughout the duration of the plateau. Similar results were seen in all five interneurons studied, as illustrated by the integral of the membrane potential change following stimulation (Figure 5D), which showed a significant increase from 5±14 mV · s (mean ± SEM) in control to 630±100 mV · s (mean ± SEM) in the presence of muscarine (*p* = 0.003, Student *t*-test). High-intensity stimulation was required to evoke the plateau, whereas low-intensity stimulation was ineffective (unpublished data). Although prolonged plateaus have not been described previously in dLGN interneurons, similar sustained elevations in membrane potential occur in other types of cells, and in some cases, they have been shown to be regulated by activation of muscarinic receptors [49].

Muscarine also had unexpected effects on the inhibitory currents recorded in TC neurons. In control conditions, there were many spontaneous inhibitory postsynaptic currents (IPSCs) observed in individual trials (representative experiment shown in Figure 5E, left, gray traces). OT stimulation evoked short-latency inhibition as has been described previously, but there was no sustained increase in the average IPSC (Figure 5E, left, black trace). In the presence of muscarine, the spontaneous inhibitory input was greatly reduced (Figure 5E, right, red traces prior to stimulation), and following OT stimulation, there was a long-lasting elevation in the number of inhibitory events, as seen in individual trials (Figure 5E, right). Muscarine consistently produced a sustained component of inhibition in these experiments and increased the inhibitory synaptic charge following stimulation from 13±9 pC (mean ± SEM) in control to 160±20 pC (mean ± SEM) in the presence of muscarine (*n* = 10, *p* = 0.0001, Student *t*-test, Figure 5F). This sustained component of inhibition was blocked by AFDX (Figure S6), indicating that it was mediated by activation of M2 receptors.

**Figure 6:**
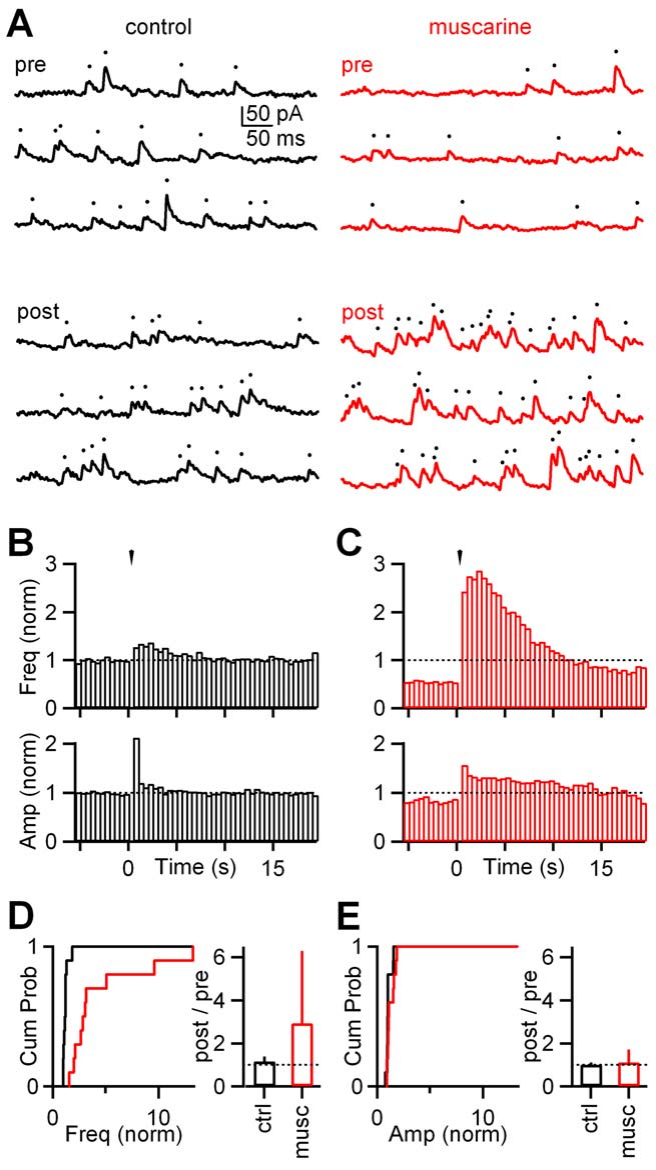
Muscarinic modulation of spontaneous and stimulus-evoked feedforward inhibition is reflected primarily in the frequency of inhibitory events. (A) Representative recording of a TC neuron performed in voltage clamp, in which spontaneous IPSCs (sIPSCs) were registered preceding (pre) and following (post) a stimulus train (five at 10 Hz). In control conditions the stimulus train did not evoke long-lasting changes in either frequency or amplitude of events (left, black traces), while in the presence of muscarine, accompanied by a decrease in frequency prior to stimulation, the same stimulus triggered a sustained increase in the number of IPSCs (right, red traces). Dots indicate timing of individual inhibitory events. (B and C) Peristimulus time histograms summarizing 10 experiments in which both frequency and amplitude of IPSCs were quantified and plotted, before (B) and after wash-in of muscarine (C). Arrowheads indicate timing of stimulus train. In (C), note the initial decrease in frequency of sIPSC and the remarkable long-lasting elevation in the frequency of evoked inhibitory events. The increase in amplitude was less pronounced. All data in (B) and (C) are normalized to frequency (freq, [B and C], top) and amplitude (amp, [B and C], bottom) values observed in control conditions prior to stimulation, respectively. (D) Left, cumulative histogram comparing post-stimulus sIPSC frequencies in control (ctrl, black) and muscarine (musc, red) conditions (*n* = 10). Notice that in the presence of muscarine, there is a marked shift toward higher frequencies. Right, a summary of 10 experiments showing that muscarine promotes a pronounced increase in the frequency of inhibitory events. Cum Prob, cumulative probability. (E). Left, cumulative histogram comparing poststimulus sIPSC amplitudes in control (black) and muscarine (red) conditions (*n* = 10). Right, muscarine-induced changes in amplitudes were modest as shown in a summary of 10 experiments. All data in (D) and (E) are normalized to frequency and amplitude values observed prior to stimulation, respectively. Data in right panels of (D) and (E) are expressed as median + IQR.

In order to better understand muscarinic modulation of spontaneous and stimulus-evoked inhibition, we performed a more detailed analysis of IPSCs in TC neurons. In control conditions, the frequency and amplitude of spontaneous IPSCs (sIPSCs) were 31±6 Hz (mean ± SEM) and 14±1 pA (mean ± SEM), respectively (*n* = 10). Brief trains (five stimuli at 10 Hz) did not evoke large sustained changes in either the frequency (mean ± SEM: 1.2±0.1 of control, *n* = 10) or amplitude (mean ± SEM: 1.1±0.1 of control, *n* = 10) of events in the 1–6-s interval following the onset of stimulation (Figure 6A, left, 6B). In the presence of muscarine, the sIPSC frequency was strongly reduced (mean ± SEM: 0.46±0.07 of control, *n* = 10, *p*<0.0001, Student *t*-test), and the amplitude was only slightly reduced (mean ± SEM: 0.84±0.06 of control, *n* = 10, *p* = 0.03, Student *t*-test; Figure 6A, right, and 6C). OT stimulation led to a prominent sustained increase in the IPSC frequency (median: 2.9-fold increase relative to control with an IQR of 3.4, *n* = 10, *p* = 0.002, Wilcoxon signed-rank test; Figure 6C, top, and 6D) and a modest increase in the IPSC amplitude (median: 1.1-fold increase relative to control with an IQR of 0.6, *n* = 10, *p* = 0.02, Wilcoxon signed-rank test; Figure 6C, bottom, and 6E). This indicates that the enhanced and sustained inhibition following stimulation of the OT with a train in the presence of muscarine arises mainly from an increase in the frequency of inhibitory events. Also, this much more pronounced change in the frequency suggests that the modulatory actions of muscarine are primarily on the presynaptic dendritic protrusions of dLGN interneurons.

### Properties of Plateau Potentials in LGN Interneurons

**Figure 7:**
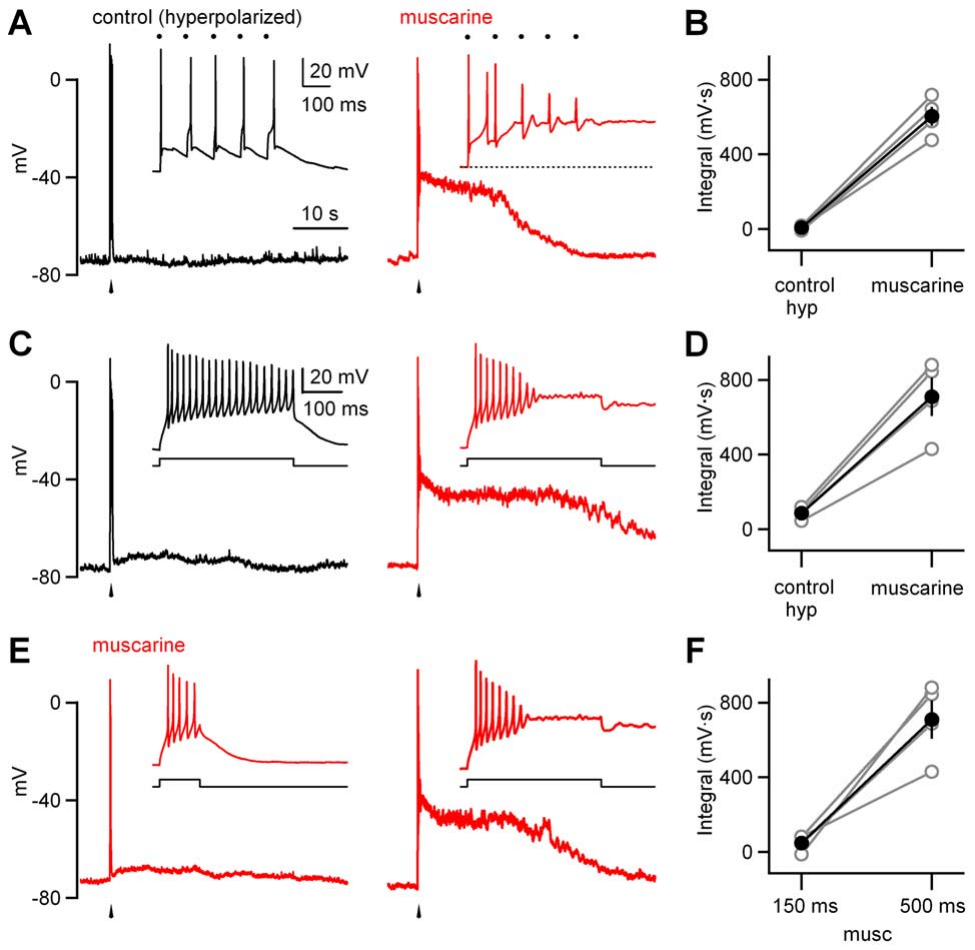
The role of initial membrane potential and depolarization in the initiation of plateau potentials in dLGN interneurons. A representative experiment is shown in which responses of dLGN interneurons were evoked in the absence of muscarine, but at a hyperpolarized (hyp) membrane potential ([A and C], left black trace) and in the presence of muscarine at resting membrane potential ([A and C], right red trace). Responses were triggered by a train of OT stimulation (five stimuli at 10 Hz, [A and B]), and by injecting 100 pA into the soma for 500 ms (C and D). OT stimulation or transient depolarization did not evoke a plateau in control conditions. (E) To test whether a shorter, more transient depolarization can trigger the prolonged plateau potential, 100 pA was injected into the soma of dLGN interneurons for 150 ms (left representative trace) and 500 ms (right representative trace). In the presence of muscarine, a depolarization of 150 ms evoked no prolonged response, but in the same cell, a 500-ms somatic depolarization evoked a long-lasting plateau potential. Insets show the responses on a finer time scale. Experiments as in (A, C, and E) are summarized in (B, D, and F), respectively. Scale bar in the inset of (C) applies to (E), time scale bar in (A) applies to (C) and (E).

We have previously shown that muscarine promotes the generation of calcium spikes primarily by hyperpolarizing dLGN interneurons (Figure 1). We therefore examined the role of hyperpolarization in the generation of the sustained plateau potential and tested whether plateau potentials could be evoked in control conditions when interneurons were hyperpolarized. OT stimulation (five stimuli at 10 Hz) did not evoke a plateau in control conditions, even when the cell was initially hyperpolarized by injecting negative current (representative experiment shown in Figure 7A, left). In the presence of muscarine, the same stimulation evoked a plateau potential even though the initial membrane potential of the cell was the same (Figure 7A, right). This was a consistent finding as indicated by the integral of the membrane potential change following stimulation, which was 7±5 mV · s (mean ± SEM) in control and 600±50 mV · s (mean ± SEM) in the presence of muscarine (*n* = 4, *p* = 0.001, Student *t*-test, Figure 7B). This indicates that factors other than hyperpolarization are involved in promoting the synaptically evoked initiation of plateau potentials in the presence of muscarine.

We went on to examine the role of depolarization in initiating prolonged plateaus. As shown in a representative cell, in control conditions, a somatic depolarization of 500 ms did not trigger a plateau potential, even when the cell was cell was initially hyperpolarized to −80 mV (Figure 7C, left), but the same depolarization triggered a prolonged plateau in the presence of muscarine (*n* = 4, *p* = 0.006, Student *t*-test, Figure 7C, right). These consistent findings (Figure 7D) indicate that somatic depolarization alone is sufficient to trigger plateaus in the presence of muscarine but not in control conditions.

One of the interesting aspects of the prolonged plateaus is that they are effectively evoked by moderate-intensity trains but not by strong single stimuli. We therefore tested whether the ability to generate long-lasting plateaus depends upon the duration of the depolarization. We found that in the presence of muscarine, a depolarization of 150 ms did not trigger a prolonged plateau (Figure 7E, left), but in the same cell, a 500-ms somatic depolarization evoked a plateau potential lasting more than 30 s (Figure 7E, right). This was a consistent finding in the presence of muscarine, with the integral of the membrane potential change following 150 ms and 500 ms depolarization being 48±22 mV · s (mean ± SEM) and 700±100 mV · s (mean ± SEM), respectively (*n* = 4, *p* = 0.01, Student *t*-test, Figure 7F). These experiments indicate that prolonging the duration of initial somatic depolarizations promotes these sustained plateau potentials in the presence of muscarine. This may explain why single stimuli are ineffective at triggering a long-lasting plateau, even if they evoke a calcium spike lasting a few hundred milliseconds, and why trains of five stimuli are able to depolarize the cell for a sufficiently long period of time to initiate it.

**Figure 8:**
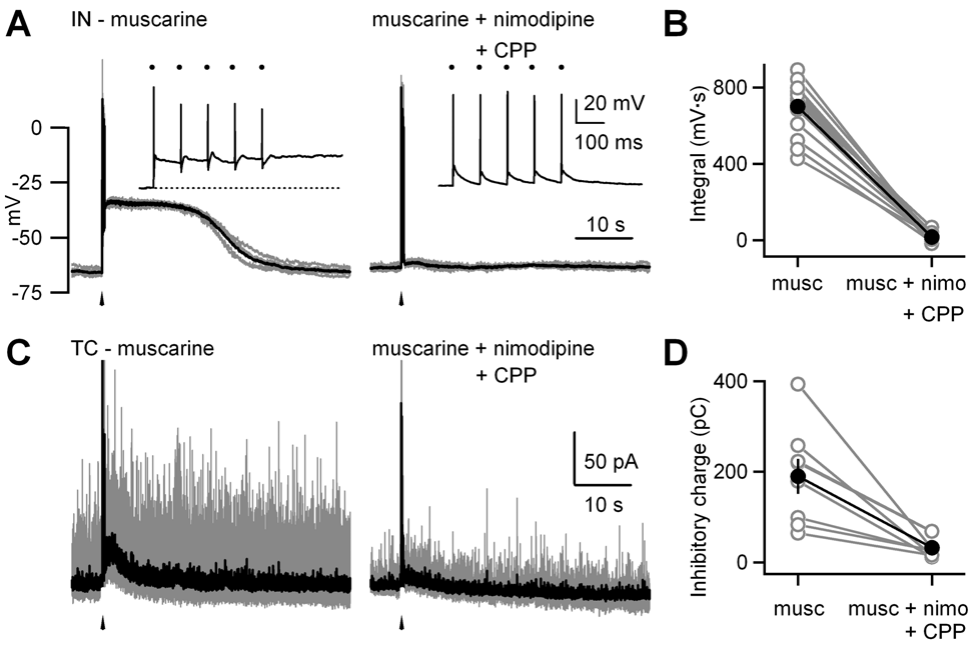
Synaptically evoked plateau potentials in dLGN interneurons and increased disynaptic inhibition in TC neurons depend upon L-type calcium channels and NMDA receptors. The OT was activated with a train of five stimuli at 10 Hz in the presence of muscarine (musc) alone (2 µM) and with addition of blockers of L-type calcium channels (10 µM nimodipine [nimo]) and NMDA receptors (5 µM R-CPP). Responses were measured in dLGN interneurons (IN) in current clamp (A and B) and in TC neurons in voltage clamp (C and D). Representative experiments in (A and C), show consecutive traces (gray) and their corresponding averages (black). Insets: evoked action potentials are shown on an expanded time scale. Arrowheads indicate timing of stimulus train and dots indicate timing of individual electrical stimuli. (B) Summary of experiments described in (A) (*n* = 15). (D) Summary of experiments described in (C) (*n* = 8).

These findings also predict that the initial transient depolarization following synaptic activation plays a key role in triggering prolonged plateaus. It is known that the transient calcium spikes following single stimuli are eliminated by blocking L-type calcium channels and NMDA receptors. We therefore tested the role of these channels in synaptic activation of the plateau potential in interneurons, and the sustained component of disynaptic inhibition seen in TC neurons following OT stimulation (five stimuli at 10 Hz). These experiments were performed in the continuous presence of muscarine. The blockade of L-type calcium channels or NMDA receptors alone had somewhat variable effects. In a number of cases, bath application of either drug alone abolished the plateau (Figure S7), but in some instances, nimodipine (Figure S7A–S7C) or CPP (Figure S7D–S7F) had only subtle effects and coapplication of both was required to eliminate the plateau (Figures S7 and 8A). Blockade of L-type calcium channels and NMDA receptors together decreased the integral of the membrane potential change following stimulation from 700±35 mV · s (mean ± SEM) to 15±6 mV · s (mean ± SEM) (Figure 8B, *n* = 15, *p*<0.0001, Student *t*-test). It also eliminated the sustained component of disynaptic inhibition (Figure 8C), and reduced evoked change in synaptic charge following stimulation from 190±40 pC (mean ± SEM) to 28±7 pC (mean ± SEM) (Figure 8D, *n* = 8, *p* = 0.001, Student *t*-test). This indicates that L-type calcium channels and NMDA receptors promote the generation of the sustained plateau in interneurons and the corresponding persistent component of disynaptic inhibition in TC cells.

## Discussion

Our main finding is that feedforward inhibition in TC neurons can be modulated in a manner that depends upon the number of activated RGCs. Muscarine greatly reduces the amplitude of disynaptic inhibition when only a few convergent RGC axons are stimulated, but when many RGC axons are synchronously activated, the time course of inhibition is slowed, and the overall stimulus-evoked inhibition increases. The key factor determining this differential modulation appears to be the ability of muscarine to distinctly control interneuron firing by either suppressing synaptically evoked interneuron spiking or by promoting the generation of long-lasting calcium spikes and prolonged plateau potentials. Thus, the regulation of firing modes allows context-dependent modulation of inhibition.

### The Mechanism Underlying Context-Dependent Modulation

Metabotropic M2 ACh receptors on the soma and dendrites of dLGN interneurons [32] are well situated to detect ACh released from cholinergic inputs arising in the brainstem parabrachial region that synapse onto the dendrites of dLGN interneurons. These synapses are often present at triads, which consist of RGC synapses onto the dendrites of a TC neuron and a dLGN interneuron, and a GABAergic synapse from the interneuron dendrite onto the TC neuron dendrite [50]. Activation of M2 receptors has been shown to decrease the frequency of both spontaneous IPSCs and miniature IPSCs recorded in TC neurons, suggesting that M2 receptors can control GABA release from dLGN interneuron dendrites [35].

Muscarinic modulation of disynaptic inhibitory responses evoked by OT stimulation has not been examined previously. Here, we find that M2 receptor activation can regulate disynaptic inhibition in complex ways that can be largely explained by the active responses of dLGN interneurons. Muscarine strongly suppresses disynaptic inhibition evoked by a single OT stimulation that in control conditions is slightly above threshold to trigger an active response in interneurons. This suppression is largely a consequence of opening potassium channels [33],[34], which both hyperpolarizes dLGN interneurons and lowers their input resistance, thereby making it more difficult to trigger spikes. As a result, low-intensity OT stimulation is unable to trigger either sodium or calcium spikes in most interneurons. Thus, changes in interneuron excitability appear to be sufficient to account for the dramatic suppression of disynaptic inhibition produced by M2 receptor activation when only a few RGC axons are activated.

For strong OT stimulation, the effects of muscarine and the mechanism of modulation are more complex. This is clearly illustrated by considering the consequences of a single, strong OT stimulation on the active responses of dLGN interneurons and the resulting disynaptic inhibition in TC neurons. In contrast to the strong suppression of inhibition evoked by weak stimulation, we find that muscarine does not reduce the overall disynaptic inhibition evoked by a single strong OT stimulation, but does prolong the time course of this inhibition. These effects on disynaptic inhibition appear to be largely a consequence of active responses in dLGN interneurons being highly sensitive to the resting membrane potential. Depolarized dLGN interneurons respond to strong OT stimulation with a single, short-latency spike, whereas hyperpolarized cells respond with multiple sodium spikes riding on a calcium action potential. In control conditions, the membrane potential of dLGN interneurons can vary, and some cells respond with a single spike, whereas others respond with a burst. By hyperpolarizing interneurons, muscarine decreases the fraction of neurons that respond to strong OT stimulation with a single short-latency spike and increases the fraction of neurons that respond with a burst of sodium spikes riding on a calcium spike. Because the IPSC observed in TC neurons is the summed response from multiple dLGN interneurons, the greater tendency for dLGN interneurons to fire calcium action potentials and bursts of sodium spikes accounts for the prolongation of the time course of the inhibition in TC neurons. When OT stimulation is sufficiently strong to overcome the increase in threshold for an active response in dLGN interneurons, the strong suppressive effects of muscarine on disynaptic inhibition are no longer observed.

The effects of muscarine on the inhibition evoked by trains of stimuli are striking. Again, there is a strong dependence on the number of RGCs that are activated. In control conditions, weak OT stimulation with brief trains evokes primarily short-latency inhibition that is reduced by muscarine. Moderate-intensity OT stimulation with a brief train leads to only brief increases in inhibition in control conditions, but elevates inhibitory activity for tens of seconds in the presence of muscarine. This remarkably long-lasting elevation in disynaptic inhibition also appears to be a consequence of the active responses of dLGN interneurons in which moderate-intensity stimulation of the OT with a brief train evokes prolonged plateaus lasting tens of seconds. Experiments in which the effects of somatic depolarization were examined revealed that although 500 ms can reliably trigger a prolonged plateau in dLGN interneurons, brief depolarizations are less effective. For this reason, the responses evoked by single strong stimuli, even those that evoke calcium spikes in the presence of muscarine, are unable to trigger prolonged plateaus. It is likely that hyperpolarization and the accompanying enhanced ability to trigger calcium spikes provides the depolarization required for OT stimulation to trigger a prolonged plateau. However, in control conditions, artificially hyperpolarizing interneurons does not effectively promote the initiation of prolonged plateaus. This suggests that muscarine modulates additional ion channels to facilitate the generation of prolonged plateaus. As a result, activation of a moderate number of RGCs, even with a brief train, is sufficient to alter the output mode of dLGN interneurons and thereby dynamically regulate the properties of feedforward inhibition.

Thus, it appears that dLGN interneurons have three distinct output modes in the presence of muscarine. First, for low-intensity stimulation, they are not effective at inhibiting TC neurons. Second, occasional high-intensity stimulation can evoke a calcium spike and sodium spikes, leading to transient inhibition of TC neurons. Finally, a brief moderate-intensity train is sufficient to shift the interneuron into an entirely different mode in which inhibition is elevated for many seconds.

### Comparison of Modulation in the dLGN to Other Brain Regions

The modulation of inhibition we observe in the dLGN is very different than the modulation that has been described in the cortex and the hippocampus [12]. In those brain regions, many different types of interneurons provide specialized inhibition to pyramidal cells. Each type of interneuron targets specific regions, exhibits different use-dependent plasticity, and responds in distinctive ways to neuromodulators [12]. In contrast, with no such diversity of interneurons within the dLGN, it is necessary for a single type of dLGN interneuron to mediate all aspects of inhibition.

In the hippocampus and cortex, activation of muscarinic receptors can selectively regulate inhibition from specific types of cells through multiple mechanisms. Muscarine hyperpolarizes some types of interneurons and depolarizes others, thereby enhancing or suppressing inhibition from different types of interneurons [51]–[57]. Inhibition can also be suppressed by activation of presynaptic M2 receptors, but only in a subpopulation of neurons that contain M2 receptors in their presynaptic boutons [58]. Lastly, muscarine can selectively regulate inhibition indirectly by activating postsynaptic receptors to promote endocannabinoid release that in turn retrogradely suppresses transmission by activating presynaptic cannabinoid CB1 receptors, which are only present on presynaptic terminals in a subset of interneurons [57],[59]–[61]. Thus, there are many mechanisms that allow muscarine to selectively regulate inhibition from different cell types in the cortex and the hippocampus. In contrast, within the dLGN muscarine regulates disynaptic inhibition provided by a single type of interneuron, and achieves spatial and temporal specificity by controlling the output mode of interneurons in a context-dependent manner.

A distinctive aspect of dLGN interneurons is that hyperpolarization can, in some circumstances, actually enhance the inhibition they provide. The key specialization is that hyperpolarization promotes the generation of calcium spikes. Previously, we have shown that calcium spikes are restricted to the dendrites, where they promote GABA release [15]. It is likely that a type of L-type calcium channel that is activated at relatively negative potentials plays an important part in mediating calcium spikes when the interneuron is hyperpolarized because a combination of L-type calcium channel antagonists and NMDA receptor antagonists eliminates calcium spikes following single stimuli [15]. T-type calcium channels have an appropriate voltage dependence to contribute to synaptically-evoked spiking in dLGN interneurons [26], but the lack of a selective antagonist makes this difficult to assess.

Muscarine also promotes afterdepolarization and sustained activity in other types of neurons, many of which are interneurons [53],[62]–[67]. Within different cells, muscarinic receptor–mediated sustained activity varies from less than a second to minutes, is mediated by different receptors, and involves diverse mechanisms (although the calcium-activated nonselective cation current *I*_CAN_ is usually involved). The prolonged plateau potentials in dLGN interneurons are of special interest because they are particularly long lasting, suggesting that the state of the interneuron is modulated for tens of seconds. Further, the depolarization is not accompanied by sustained spiking. Although similar sorts of plateaus have also been seen in other cell types [68],[69], they are of particular interest in dLGN interneurons, which can release GABA from both dendrites and axons [15]. Because we have previously shown that calcium spikes are restricted to the dendrites [15], it appears that the prolonged plateau results in functional segregation in which GABA is preferentially released from the dendrites and not from the axon.

### Implications for Modulating Light-Evoked Responses in the dLGN

Visually evoked responses of the dLGN are highly dependent on behavioral state. For instance, arousal from sleep has been shown to promote a strong enhancement in the response of TC neurons [70],[71]. The parabrachial region (PBR) of the brainstem, which exhibits increased activity during states of alertness and attention, plays an important role in regulating visual responses in the dLGN [72],[73]. The effects of the PBR appear to be largely due to the extensive cholinergic input it provides to the dLGN [31],[74],[75]. Thus, understanding cholinergic modulation within the dLGN is of particular importance, which will provide us a more detailed insight into how behavioral state influences the way visual responses are conveyed to the cortex.

Parabrachial and cholinergic activation within the dLGN can lead to diverse and often contradictory effects on TC activity. Most studies have focused on the RFs of TC neurons, which have a center-surround organization [76] in which inhibitory interneurons in the dLGN [77]–[79] accentuate the surround inhibition. In some studies, PBR activation enhanced the relay of visual signals [80]–[84] and increased visual resolution [83] without affecting the shape of the RF [85]. Several studies have found that in conditions similar to those during activation of the PBR, visually driven responses of dLGN interneurons providing feedforward inhibition were depressed [33],[86]. In other studies, increased PBR activity, awaking from sleep, or elevated levels of ACh within the dLGN, all increased surround inhibition within TC neuron RFs [22],[70],[71],[78],[84],[87].

Our findings provide a possible explanation for how cholinergic activation provided by the PBR can have such diverse effects on TC neuron responses. The muscarinic modulation of inhibition we describe here would act in concert with the direct effect on TC neurons to regulate visually activated dLGN output. Our results suggest that for visual input that stimulates a small number of RGCs, ACh would substantially reduce inhibition provided by dLGN interneurons, thereby increasing the gain of TC neurons and making them more responsive to weak inputs. In contrast, we predict that for patterns of visual stimulation that promote synchronous activity of a high number of RGCs, ACh could increase disynaptic inhibition onto TC neurons. This context-dependent feature of feedforward inhibition in the dLGN may be an important component responsible for the dynamics of feature selectivity in vision.

## Materials and Methods

### Animals and Slice Preparation

Mice (24–32 d old, C57BL/J6) were deeply anesthetized with isoflurane or halothane; their brains were then quickly removed and placed in ice-cold oxygenated (95% oxygen/5% CO_2_) high-sucrose solution containing (in millimolar concentration): 220 sucrose, 2.5 KCl, 6 MgCl_2_, 1 CaCl_2_, 1.25 NaH_2_PO_4_, 26 NaHCO_3_, and 10 glucose. A block of brain tissue containing the OT and the dLGN was gently dissected out and transferred to a vibratome where 220–250-µm pseudosaggital slices were cut, and then moved to an equilibrium chamber filled with oxygenated artificial cerebrospinal fluid that contained (in millimolar concentration): 24 NaCl, 3 KCl, 2 MgCl_2_, 2 CaCl_2_, 1.23 NaH_2_PO_4_, 26 NaHCO_3_, and 25 glucose. After 1–2 h of recovery, brain slices with an intact OT and dLGN were placed in the recording chamber mounted on an Olympus BX51WI microscope equipped with DIC and fluorescence capabilities. The temperature in the recording chamber was kept near 34°C using an inline heater controller (Warner Instruments). The tissue was continuously superfused with oxygenated ACSF at approximately 5 ml/min flow rate.

### Electrophysiological Recordings and Data Analysis

The inhibitory interneurons within the dLGN were identified by selective GFP expression in C57BL/J6 GAD67-GFP transgenic animals [88], whereas for most of the TC cell recordings, wild-type C57BL/J6 animals were used. Green fluorescent protein–expressing cells within the dLGN were identified using appropriate fluorescence filters and then approached under DIC. In some experiments, loose cell–attached recordings were performed by gently placing the recording pipette (filled with normal ACSF) over the cell membrane, thereby establishing a low-resistance seal between the pipette and the cell surface (∼50–100 MΩ). A K^+^-based internal solution (see below) was used in experiments where dLGN interneuron responses were first monitored extracellularly and then in whole-cell mode. In most of the experiments, a high-resistance gigaseal was first established between the recording pipette and the cell membrane. Whole-cell configuration was achieved by the application of additional brief pulses of negative pressure through the recording pipette.

Whole-cell voltage- and current-clamp recordings were performed by using 2–3.5-MΩ borosilicate glass pipettes pulled with a Sutter horizontal puller (Sutter Instruments) and filled with an intracellular solution that contained the following (in millimolar concentration): 150 Cs-gluconate (or KMeSO_4_ for current-clamp recordings), 1 MgCl_2_, 10 HEPES, 0.5 EGTA, 2 Mg-ATP, 0.4 Na_2_-GTP, and 10 Na_2_-phosphocreatine (pH 7.3) with CsOH (or KOH for KMeSO_4_-based solution). Some current-clamp interneuron recordings were performed with an internal solution that contained (in millimolar concentration): 150 K-gluconate, 1 MgCl_2_, 5 HEPES, 1.1 EGTA, 2 Mg-ATP, 10 Na_2_-phosphocreatine (pH 7.3) with KOH. Access resistance was continuously monitored throughout the experiments, and only those cells with stable access resistance (changes <10%) were used for analysis.

The resting membrane potential of dLGN interneurons was assessed from the reversal potential of voltage-activated K^+^-currents that were activated with a voltage ramp protocol applied in a cell-attached configuration (*R*_seal_ >1 GΩ), in which pipettes were filled with a solution containing (in millimolar concentration): 145 KCl, 2 EGTA, 2 MgCl_2_, and 5 HEPES; pH was adjusted to 7.3 with ∼10 KOH. Measurements were based on the principle that with similar concentrations of K^+^ in the recording pipette and the cytoplasm (155 mM), voltage-activated K^+^-currents will reverse when the potential difference across the membrane patch is 0 mV, in other words, when the pipette potential cancels out the membrane potential. Therefore, the holding potential at which the currents reverse direction will give us a quantitative measure of the resting membrane potential (Figure S1, [36]–[38]). In all cases, prior to cell-attached recordings, the amplifier was set to zero with the recording pipette in the bath solution, to null the junction potential between the bath and the pipette solution. The recordings were performed with a 700B Axoclamp amplifier (Axon Instruments) and were controlled with custom software written in Igor (generously provided by Mathew Xu-Friedman).

Extracellular RGC axon stimulation was performed either with theta glass electrodes or with custom stainless steel Teflon-coated bipolar electrodes (tip separation ∼0.1 to 0.4 mm). Stimulation electrodes were placed in the OT at least 1 mm away from the ventral border of the dLGN. Brief (300 µs, 0–200 µA) electrical pulses were delivered through an A395 Linear Stimulus Isolator (WPI). In most experiments, synaptically evoked responses were recorded at intervals of 30 s. A subset of interneuron recordings (Figures 1–3, with the exception of Figure S3) were performed in the presence of picrotoxin (20 µM) and CGP 55845 (2 µM) to block GABA_A_ and GABA_B_ receptors, respectively. Voltage-clamp recordings of interneurons were carried out at a holding potential of −70 mV. To record disynaptic currents in TC neurons, cells were held at either −10 or 0 mV. MK-801 (2 mM) was included in the internal solution to selectively block NMDA receptors in the recorded TC cell without affecting the receptors on interneurons. In a subset of experiments (Figure 5), disynaptic inhibitory currents in TC neurons were recorded with CGP 55845 (2 µM) in the external solution.

In some experiments, the actions of muscarine were measured following low- or high-intensity OT stimulation. In all these cases, the minimal current necessary to activate RGC axons was first determined by sequentially stimulating the OT at progressively higher intensities (from 0 to 200 µA, intervals of 10–20 µA, every 30 s) while recording either from interneurons (Figure 2) or TC cells (Figure 4). With increasing intensities, EPSP/Cs increased in amplitude indicating recruitment of additional RGCs [15]. Once the threshold stimulating current was determined (∼25 µA), the stimulus intensity was kept either just above the threshold or approximately 100 µA higher than the threshold for low- and high-intensity experiments, respectively.

In order to consistently activate muscarinic receptors with a known concentration of agonist, we bath applied ACh or muscarine rather than locally applying it via pressure or iontophoresis. Desensitization of the modulation was not apparent, provided the concentration of muscarine was low (2 µM) and the duration of application was less than 5 min. Although a selective M2 receptor agonist is not available, the modulation produced by ACh or muscarine was a result of this receptor subtype activation, as it was prevented by the selective antagonist AFDX (Figures 2, 3, S5, and S6).

To reliably evoke and study plateau potentials in dLGN interneurons optimal slice orientation was necessary, in which we were able to stimulate a sufficient number of RGC axons to elicit active responses. Under such conditions dLGN interneurons, that were initially hyperpolarized to ∼−70 mV, exhibited calcium spikes of >100 ms duration to single electrical stimuli delivered to the OT. To quantify the long-lasting plateau potentials in dLGN interneurons and the corresponding sustained component of inhibition in TC neurons, the integral of the membrane potential change and holding current change was calculated over 30 s following stimulation, respectively. Detection and quantification of IPSCs was carried out using custom software written in Igor 6.0 in which both the first derivative and the amplitude were used to detect events. Visual inspection was used to verify the ability to detect a very high fraction of the events while avoiding interference by noise in the current trace. First, we calculated the average frequency and amplitude of sIPSCs in control conditions prior to stimulation. Peristimulus time histograms of IPSC frequency and amplitude were then plotted using a bin size of 500 ms. Frequency and amplitude values were normalized to control values for each bin. For comparison purposes, average frequency and amplitude values were calculated over a time period of 5 s before and after the stimulus train (prestimulus: 0.5–5.5 s, poststimulus: 6–11 s). These poststimulus averages were then normalized to the corresponding prestimulus average frequency and amplitude values.

### Two-Photon Imaging

Two-photon calcium imaging experiments were performed as described previously [15],[89] using a custom two-photon laser-scanning microscope with a 60× objective (either 0.9 or 1.1 numerical aperture, NA) (Olympus Optical) and a Ti:sapphire laser (Mira; Coherent). dLGN interneurons were loaded with the calcium indicator Fluo 5F (50 µM) and the fluorescent dye Alexa 594 (50 µM) for visualization of interneuron processes. A single excitation wavelength of near 810 nm was effective for simultaneous excitation of both green and red fluorophores. Calcium elevations are expressed as the ratio of the change in the green and red fluorescence (Δ*G*/*R*). For all imaging experiments, calcium indicators were dissolved in a current-clamp internal solution containing (in millimolar concentration) 150 KMeSO_4_, 1 MgCl_2_, 10 HEPES, 2 Mg-ATP, 0.4 Na_2_-GTP, and 10 Na_2_-phosphocreatine (pH 7.3) with KOH.

### Statistical Comparisons

All data were subject to Shapiro-Wilk *W* normality test prior to statistical comparisons. Depending on whether the given dataset was normally distributed or not (*p* = 0.01), a Student paired *t*-test or a Wilcoxon signed-rank test was performed, respectively. For the comparison of more than two groups, a parametric ANOVA or a nonparametric K-W ANOVA was carried out, respectively, followed by a post hoc LSD test. All data are expressed as mean ± SEM or median ± IQR, respectively.

## Supporting Information

Figure S1 **Cell-attached measurement of resting membrane potential of dLGN interneurons.** Depolarizing voltage ramps with a duration of 137.5 ms were applied to dLGN interneurons in cell-attached configuration with a seal resistance of >1 GΩ (top). Average of 10 consecutive current traces recorded during the voltage ramp is shown (black, bottom). The dotted red line is the extrapolated leak current from a linear fit of the initial portion (approximately 50-ms window, from 20 ms after the start of the ramp) of the average current trace. The vertical dotted black line indicates the intersection of the voltage-activated K^+^-current with the leak current, yielding the resting membrane potential of the recorded cell (arrow), the pipette potential (*V*_pipette_) at which the current reverses.
(0.41 MB TIF)

Figure S2 **Muscarine does not affect synaptic transmission between RGCs and dLGN interneurons.** Excitatory postsynaptic currents were evoked in dLGN interneurons in voltage clamp by activation of RGC axons with a wide range of stimulus intensities from low to high. (A) Representative averaged EPSCs evoked by a pair of high-intensity stimuli in control conditions (black) and in the presence of muscarine (red) are shown. Stimulus artifacts were digitally removed for clarity. Dots indicate timing of stimuli. (B and C) Summary of nine experiments in which the actions of muscarine on initial EPSC amplitude (B) and paired-pulse plasticity (C) were studied. Muscarine had no effect on either amplitude (*p* = 0.66) or paired-pulse ratio (*p* = 0.06). (D and E) There was also no correlation between the extent of the muscarine effect on amplitude (D) or paired-pulse ratio (E) and the initial EPSC amplitude. Evoked EPSCs ranged from being subthreshold to suprathreshold of action potential initiation.
(0.67 MB TIF)

Figure S3 **Intact GABAergic signaling does not influence muscarinic actions on dLGN interneuron output.** Additional experiments were conducted in which the OT was stimulated and the resulting dLGN interneuron firing was monitored with an on-cell electrode in the absence of picrotoxin and CGP55845. This allowed us to noninvasively monitor the effect of muscarine on the responses of dLGN interneurons with intact GABAergic signaling. (A) Traces from a representative experiment before and after bath application of muscarine. Stimulus artifacts were digitally removed for clarity. Dots indicate timing of stimuli. (B and C) Cumulative histograms (*n* = 28 cells, five trials per cell for each experimental condition) show the distribution of cells in which a given number of spikes were evoked (B) and the average number and timing of spikes evoked per trial (C) in control conditions (black), in the presence of muscarine (red) and after washout (gray).
(0.50 MB TIF)

Figure S4 **Muscarine enhances dendritic calcium transients triggered by synaptic stimulation.** Recordings were made from dLGN interneurons with a pipette containing the green calcium indicator Fluo 5F (50 µM) and the red dye Alexa 594 (50 µM) to visualize the regions of interest. (A) Two-photon fluorescence image of a representative dLGN interneuron with a white box indicating the dendritic region selected for imaging. (B) Electrical responses of dLGN interneurons and dendritic calcium signals evoked by identical OT stimulation in control conditions (left) and in the presence of muscarine (right). Calcium signals are expressed as the ratio of the fluorescence of the green calcium indicator (Fluo 5F) and the red calcium-insensitive dye (Alexa 594). The membrane potential was −51 mV in control conditions and −68 mV in the presence of muscarine. Arrowheads indicate timing of stimuli. (C) Summary for three experiments in which dendritic calcium elevations (expressed as the ratio of green to red fluorescence, Δ*G/R*) were measured in control conditions and in the presence of muscarine.
(0.62 MB TIF)

Figure S5 **M2 receptors in interneurons mediate muscarinic modulation of feedforward inhibition.** Synaptic currents were recorded in TC neurons while stimulating the OT at intensities of 50–100 µA. (A) Top: direct EPSCs (inward currents) and disynaptic IPSCs (outward currents) recorded in a representative experiment in control, muscarine, and AF-DX116 conditions. IPSCs were suppressed by muscarine, and this was reversed by the M2 receptor antagonist, AF-DX 116, whereas EPSCs (arrow) were unaffected by muscarine. Bottom: summary graph showing the effects of muscarine and AFDX on averaged IPSC (open bars) or EPSC (filled bars) amplitude. (B) Experiments were conducted that were similar to those in (A), but the holding potential was adjusted to allow the IPSC to be studied in isolation. In these experiments, the application of muscarine in the presence of AFDX failed to reduce disynaptic inhibition in TC cells. This indicates that muscarine suppressed transmission by activating M2 receptors. In all experiments, the application of the specific AMPA-receptor antagonist, NBQX, eliminated the outward current. This indicates that the inhibition is disynaptic and relies upon the activation of dLGN interneurons. Stimulus artifacts were removed for clarity. Arrowheads indicate timing of stimuli.
(0.41 MB TIF)

Figure S6 **Muscarine-evoked sustained inhibition is mediated by M2 receptors.** Voltage-clamp recordings were conducted in TC neurons in which the OT was activated with a train of 5 stimuli at 10 Hz, first in the presence of muscarine alone (2 µM) and then with addition of the specific M2 receptor antagonist, AFDX (10 µM). (A) In a representative experiment, consecutive current traces (light traces) and their corresponding averages (dark traces) are shown. The stimulus-evoked long-lasting increase in feedforward inhibition was eliminated by the application of the antagonist. Arrowheads indicate timing of stimulus train. (B) Summary of experiments described in (A) (*n* = 3).
(0.74 MB TIF)

Figure S7 **L-type calcium channels and NMDA receptors synergistically mediate persistent muscarine-induced activation of dLGN interneurons.** The OT was activated with a train of five stimuli at 10 Hz in the presence of muscarine alone (2 µM) and with sequential addition of blockers of L-type calcium channels (10 µM nimodipine) and NMDA receptors (5 µM R-CPP). Responses were measured in dLGN interneurons in current clamp (A, B, D, and E). Representative experiments in (A and B) and (D and E) show consecutive traces (gray) and their corresponding averages (black). Arrowheads indicate timing of stimulus train. (C) Summary of experiments as in (A and B) (*n* = 6). (F) Summary of experiments as in (D and E) (*n* = 4). Time scale bar in (A) applies to (A, B, D, and E).
(0.80 MB TIF)

## Abbreviations

dLGN: dorsal lateral geniculate nucleus
EPSC: excitatory postsynaptic current
IPSC: inhibitory postsynaptic current
IQR: interquartile range
K-W: Kruskal-Wallis
OT: optic tract
PBR: parabrachial region
RF: receptive field
RGC: retinal ganglion cell
sIPSC: spontaneous inhibitory postsynaptic current
TC: thalamocortical
