# Supplementary figures and images for: Cholinergic Activation of M2 Receptors Leads to Context-Dependent Modulation of Feedforward Inhibition in the Visual Thalamus
Source: PLoS Biol. 2010 Apr 6;8(4):e1000348. doi: 10.1371/journal.pbio.1000348 (PMC2850378; doi:10.1371/journal.pbio.1000348)

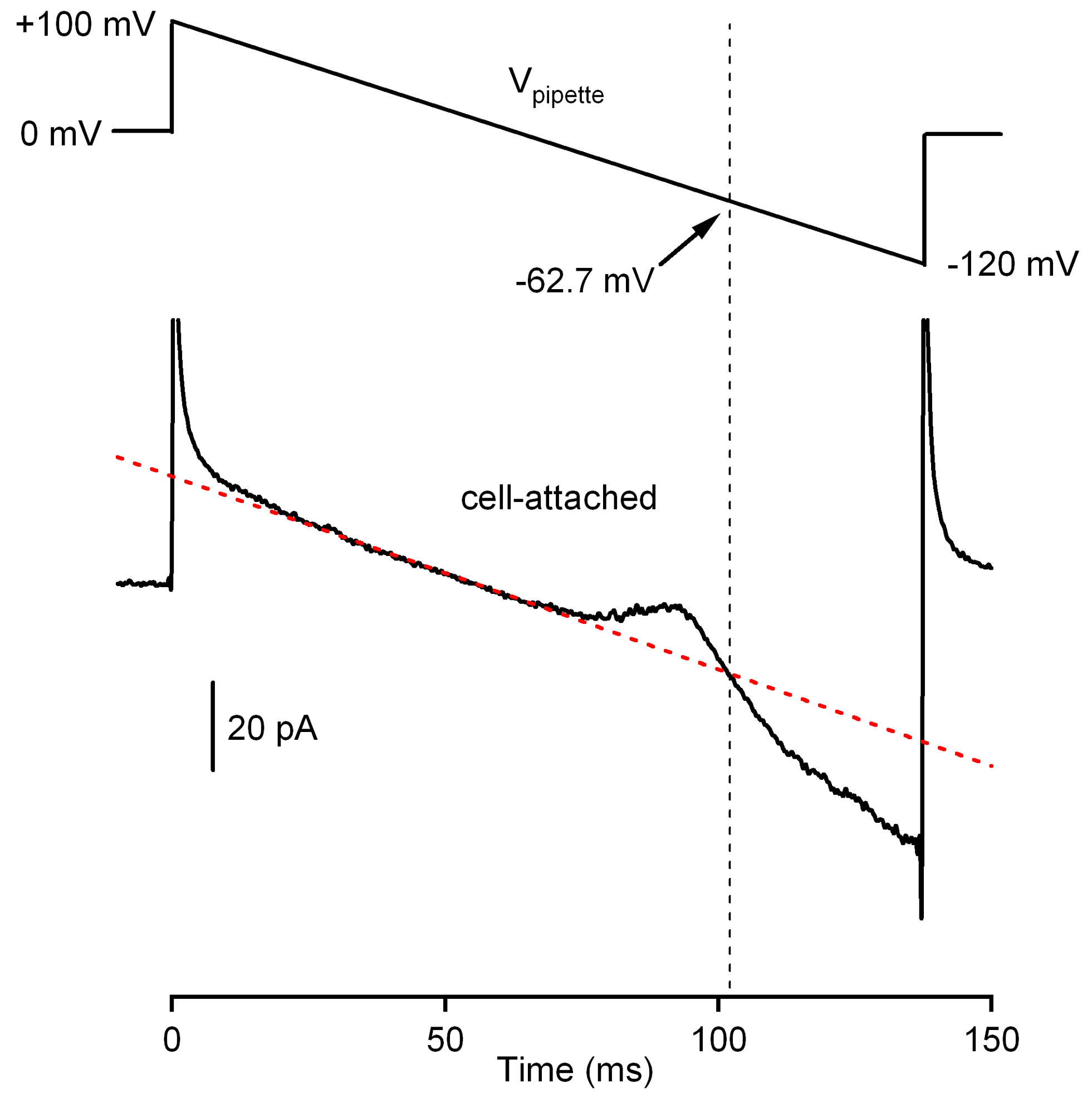

Supplement: Figure S1 — Cell-attached measurement of resting membrane potential of dLGN interneurons. Depolarizing voltage ramps with a duration of 137.5 ms were applied to dLGN interneurons in cell-attached configuration with a seal resistance of >1 GΩ (top). Average of 10 consecutive current traces recorded during the voltage ramp is shown (black, bottom). The dotted red line is the extrapolated leak current from a linear fit of the initial portion (approximately 50-ms window, from 20 ms after the start of the ramp) of the average current trace. The vertical dotted black line indicates the intersection of the voltage-activated K+-current with the leak current, yielding the resting membrane potential of the recorded cell (arrow), the pipette potential (V pipette) at which the current reverses. (0.41 MB TIF) [file pbio.1000348.s001.tif]

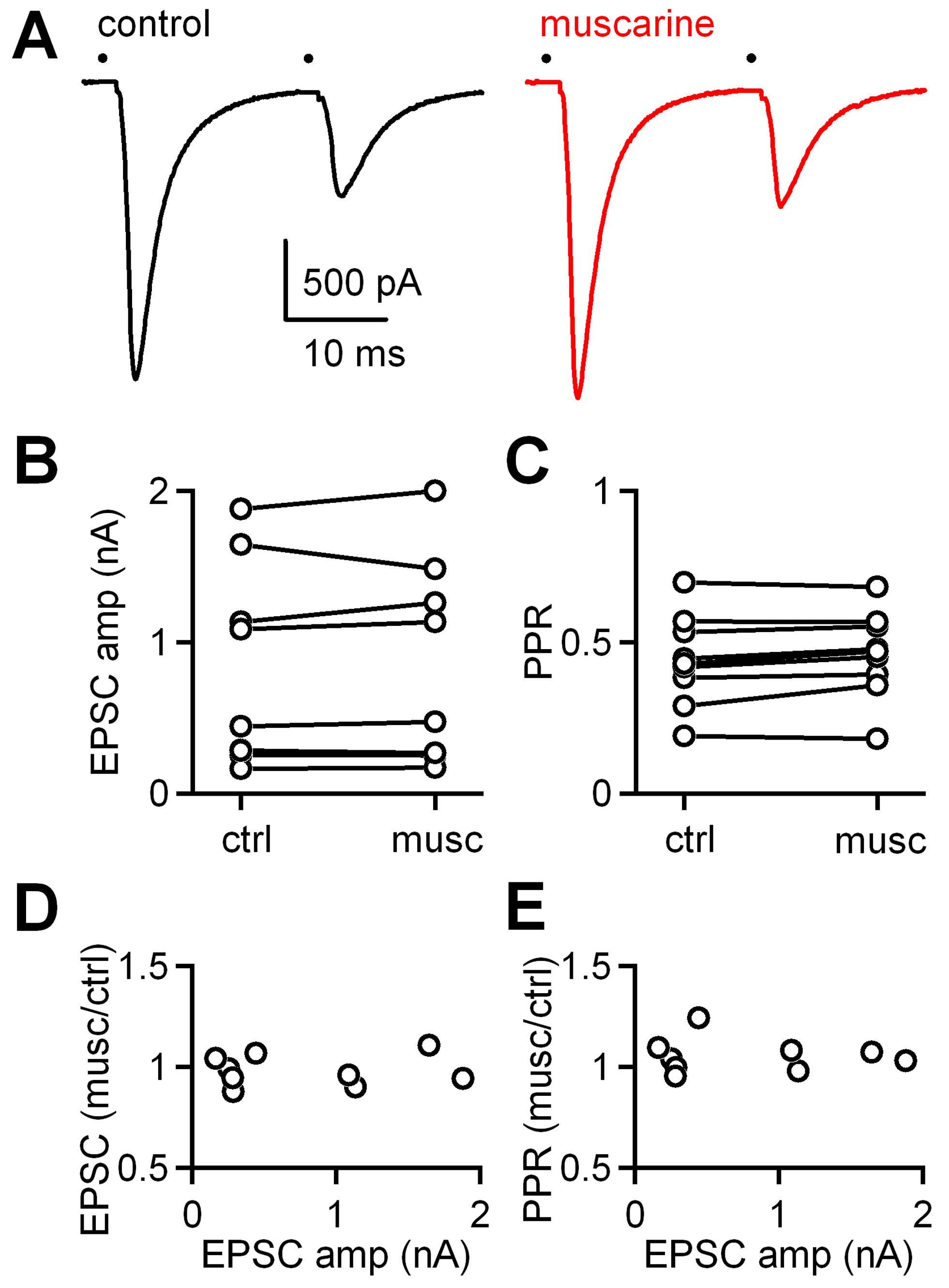

Supplement: Figure S2 — Muscarine does not affect synaptic transmission between RGCs and dLGN interneurons. Excitatory postsynaptic currents were evoked in dLGN interneurons in voltage clamp by activation of RGC axons with a wide range of stimulus intensities from low to high. (A) Representative averaged EPSCs evoked by a pair of high-intensity stimuli in control conditions (black) and in the presence of muscarine (red) are shown. Stimulus artifacts were digitally removed for clarity. Dots indicate timing of stimuli. (B and C) Summary of nine experiments in which the actions of muscarine on initial EPSC amplitude (B) and paired-pulse plasticity (C) were studied. Muscarine had no effect on either amplitude (p = 0.66) or paired-pulse ratio (p = 0.06). (D and E) There was also no correlation between the extent of the muscarine effect on amplitude (D) or paired-pulse ratio (E) and the initial EPSC amplitude. Evoked EPSCs ranged from being subthreshold to suprathreshold of action potential initiation. (0.67 MB TIF) [file pbio.1000348.s002.tif]

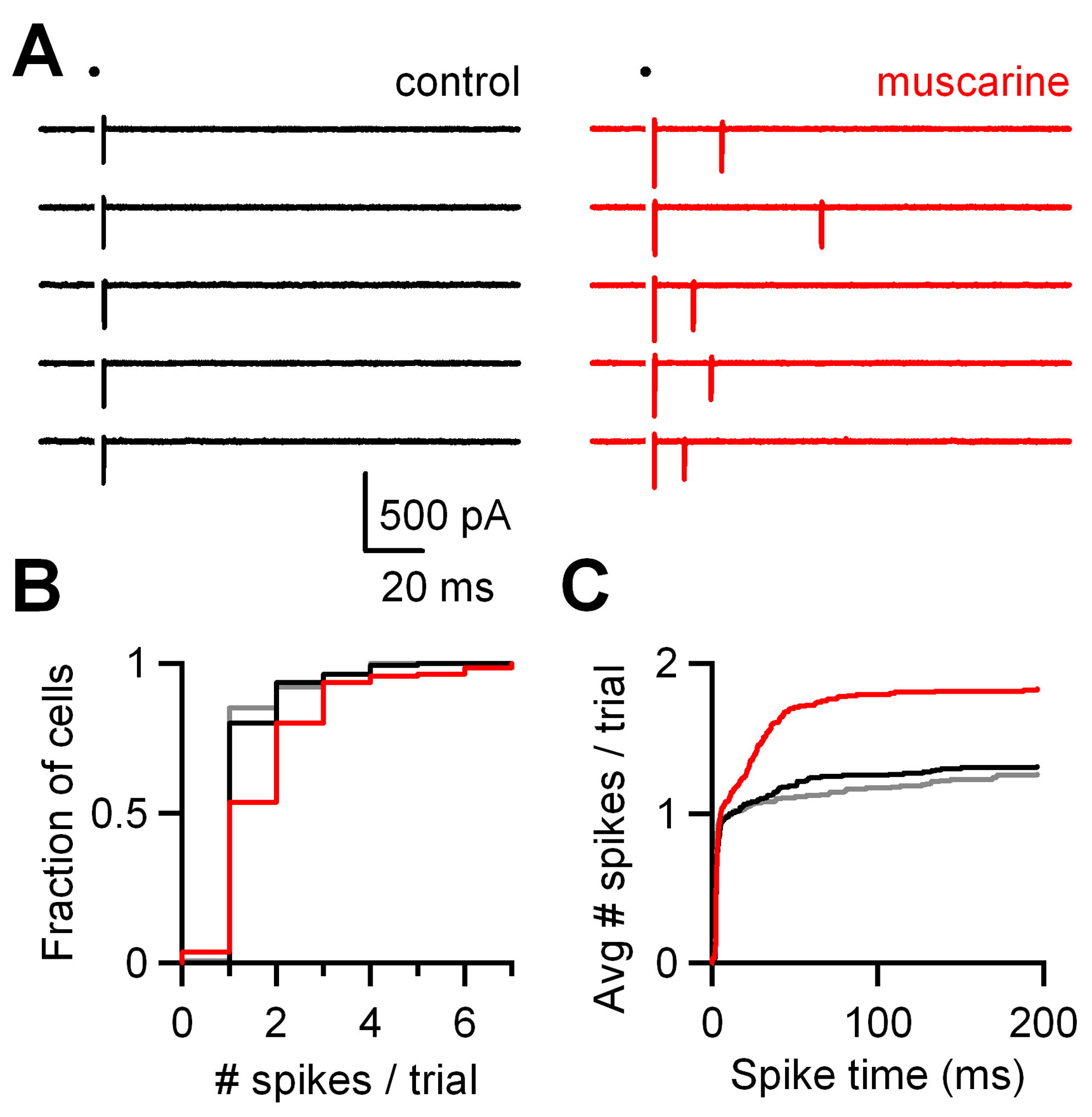

Supplement: Figure S3 — Intact GABAergic signaling does not influence muscarinic actions on dLGN interneuron output. Additional experiments were conducted in which the OT was stimulated and the resulting dLGN interneuron firing was monitored with an on-cell electrode in the absence of picrotoxin and CGP55845. This allowed us to noninvasively monitor the effect of muscarine on the responses of dLGN interneurons with intact GABAergic signaling. (A) Traces from a representative experiment before and after bath application of muscarine. Stimulus artifacts were digitally removed for clarity. Dots indicate timing of stimuli. (B and C) Cumulative histograms (n = 28 cells, five trials per cell for each experimental condition) show the distribution of cells in which a given number of spikes were evoked (B) and the average number and timing of spikes evoked per trial (C) in control conditions (black), in the presence of muscarine (red) and after washout (gray). (0.50 MB TIF) [file pbio.1000348.s003.tif]

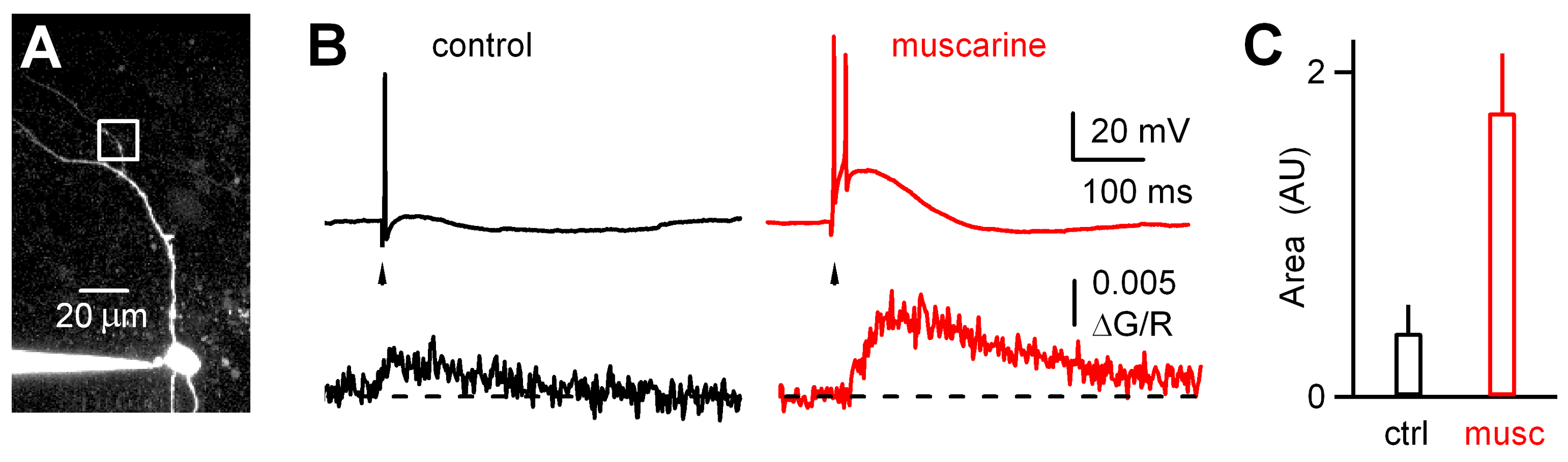

Supplement: Figure S4 — Muscarine enhances dendritic calcium transients triggered by synaptic stimulation. Recordings were made from dLGN interneurons with a pipette containing the green calcium indicator Fluo 5F (50 µM) and the red dye Alexa 594 (50 µM) to visualize the regions of interest. (A) Two-photon fluorescence image of a representative dLGN interneuron with a white box indicating the dendritic region selected for imaging. (B) Electrical responses of dLGN interneurons and dendritic calcium signals evoked by identical OT stimulation in control conditions (left) and in the presence of muscarine (right). Calcium signals are expressed as the ratio of the fluorescence of the green calcium indicator (Fluo 5F) and the red calcium-insensitive dye (Alexa 594). The membrane potential was −51 mV in control conditions and −68 mV in the presence of muscarine. Arrowheads indicate timing of stimuli. (C) Summary for three experiments in which dendritic calcium elevations (expressed as the ratio of green to red fluorescence, ΔG/R) were measured in control conditions and in the presence of muscarine. (0.62 MB TIF) [file pbio.1000348.s004.tif]

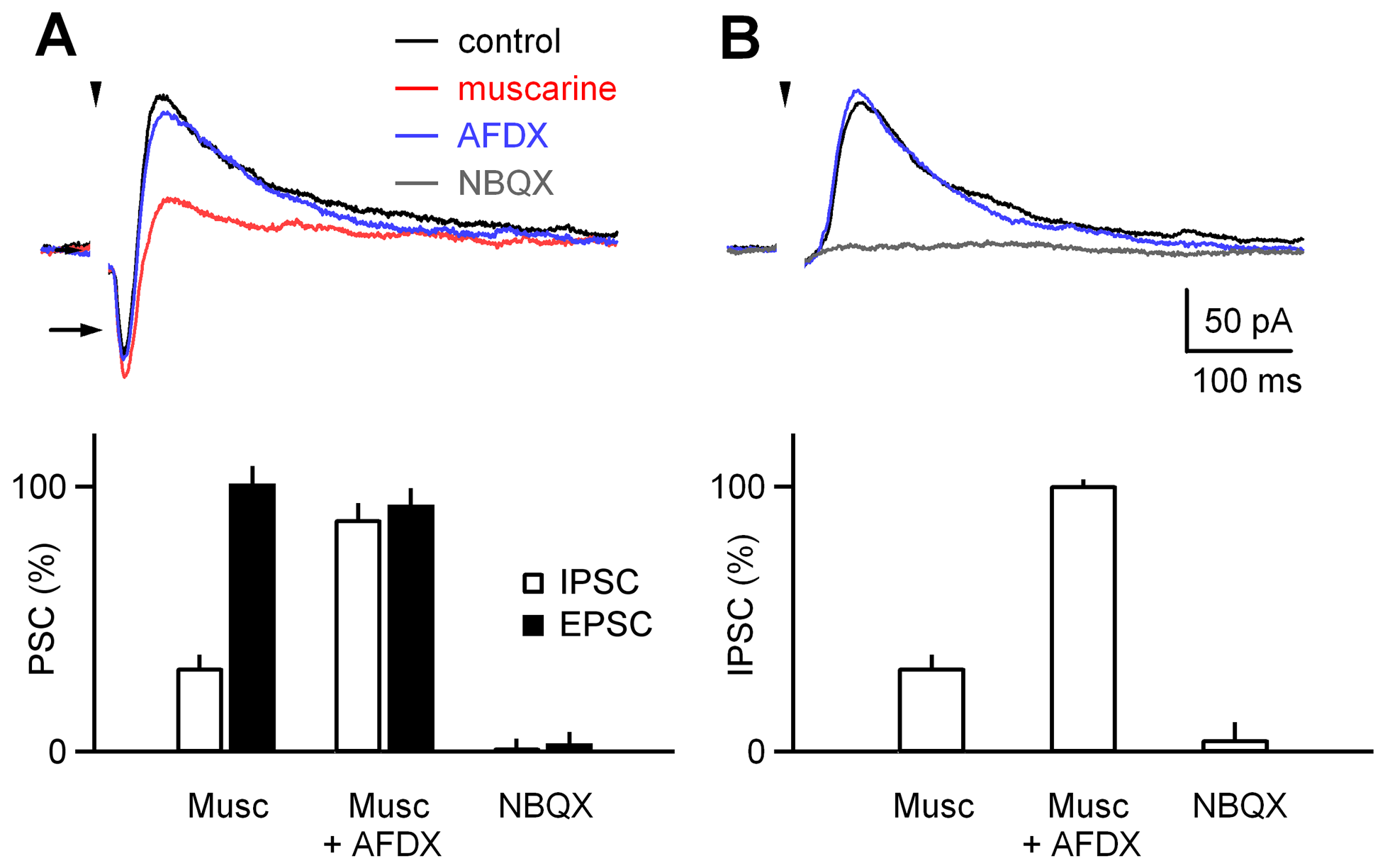

Supplement: Figure S5 — M2 receptors in interneurons mediate muscarinic modulation of feedforward inhibition. Synaptic currents were recorded in TC neurons while stimulating the OT at intensities of 50–100 µA. (A) Top: direct EPSCs (inward currents) and disynaptic IPSCs (outward currents) recorded in a representative experiment in control, muscarine, and AF-DX116 conditions. IPSCs were suppressed by muscarine, and this was reversed by the M2 receptor antagonist, AF-DX 116, whereas EPSCs (arrow) were unaffected by muscarine. Bottom: summary graph showing the effects of muscarine and AFDX on averaged IPSC (open bars) or EPSC (filled bars) amplitude. (B) Experiments were conducted that were similar to those in (A), but the holding potential was adjusted to allow the IPSC to be studied in isolation. In these experiments, the application of muscarine in the presence of AFDX failed to reduce disynaptic inhibition in TC cells. This indicates that muscarine suppressed transmission by activating M2 receptors. In all experiments, the application of the specific AMPA-receptor antagonist, NBQX, eliminated the outward current. This indicates that the inhibition is disynaptic and relies upon the activation of dLGN interneurons. Stimulus artifacts were removed for clarity. Arrowheads indicate timing of stimuli. (0.41 MB TIF) [file pbio.1000348.s005.tif]

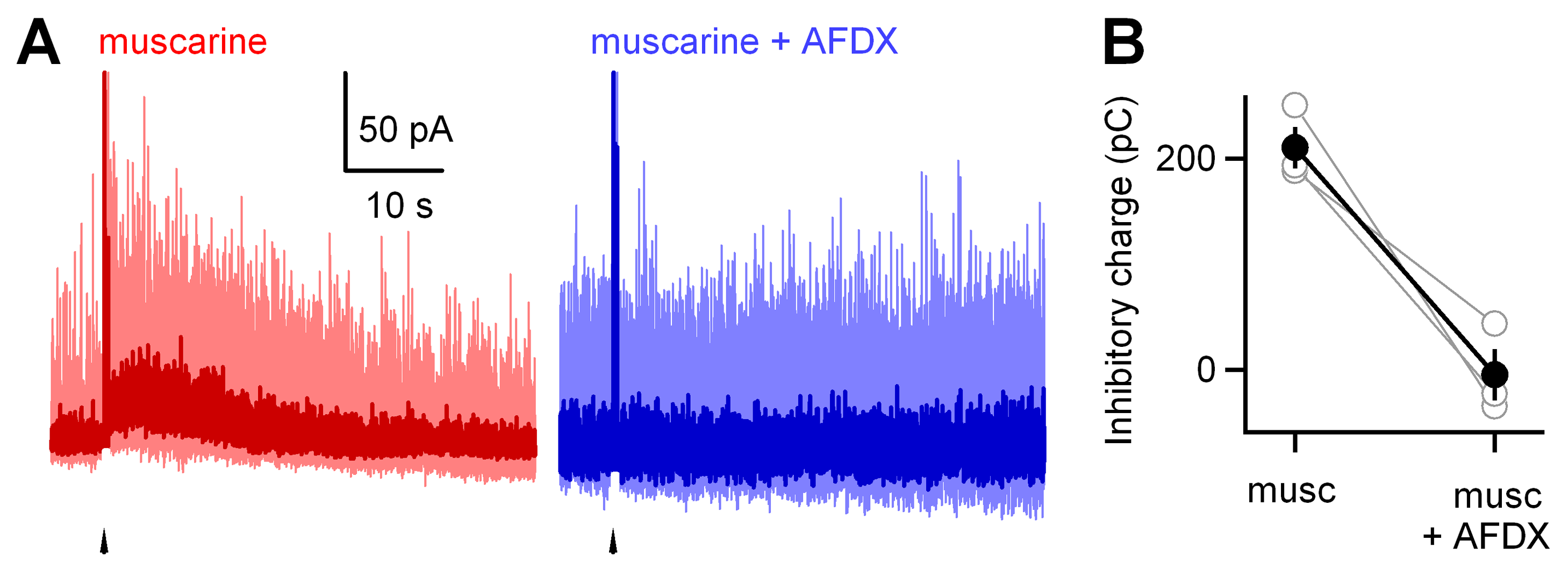

Supplement: Figure S6 — Muscarine-evoked sustained inhibition is mediated by M2 receptors. Voltage-clamp recordings were conducted in TC neurons in which the OT was activated with a train of 5 stimuli at 10 Hz, first in the presence of muscarine alone (2 µM) and then with addition of the specific M2 receptor antagonist, AFDX (10 µM). (A) In a representative experiment, consecutive current traces (light traces) and their corresponding averages (dark traces) are shown. The stimulus-evoked long-lasting increase in feedforward inhibition was eliminated by the application of the antagonist. Arrowheads indicate timing of stimulus train. (B) Summary of experiments described in (A) (n = 3). (0.74 MB TIF) [file pbio.1000348.s006.tif]

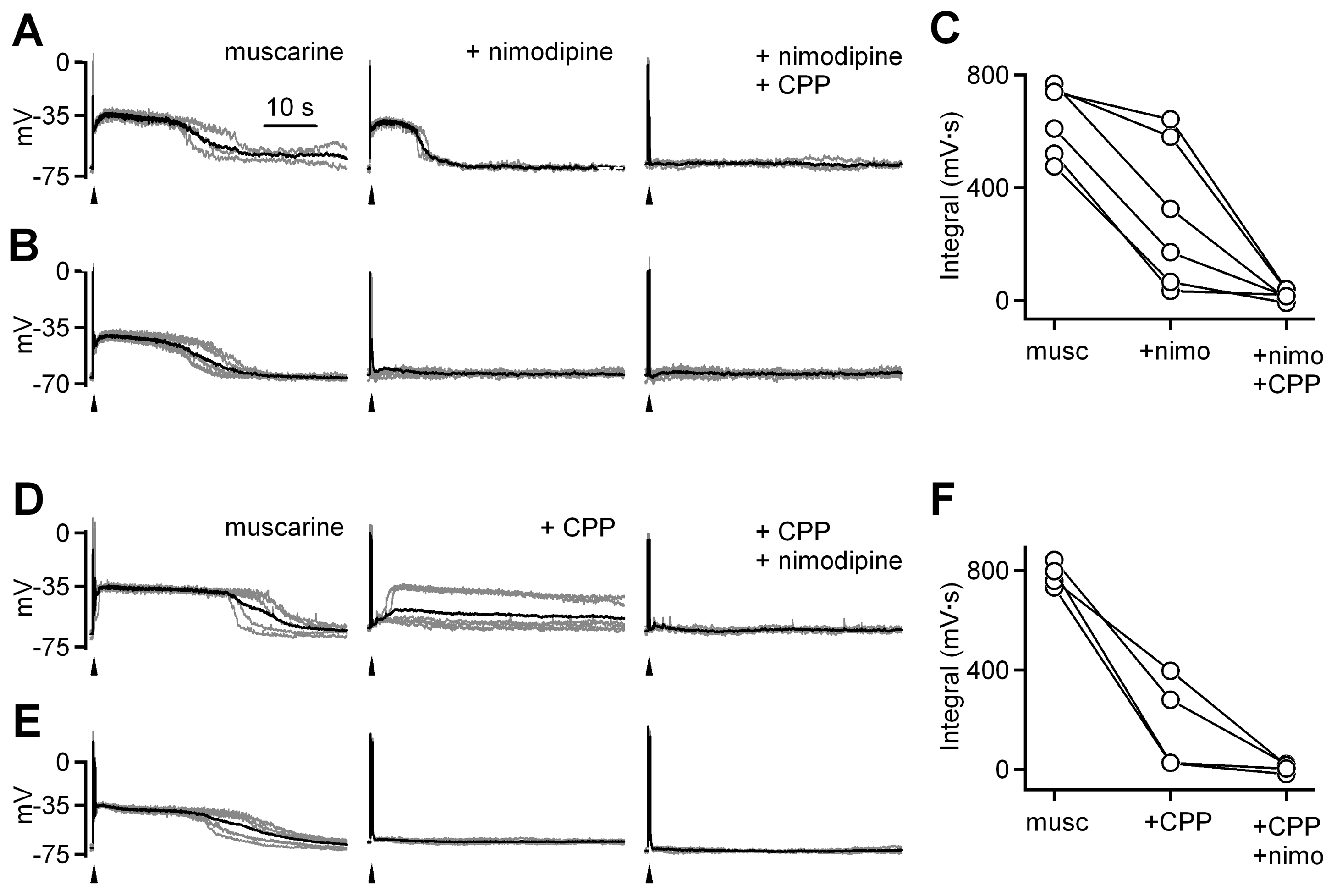

Supplement: Figure S7 — L-type calcium channels and NMDA receptors synergistically mediate persistent muscarine-induced activation of dLGN interneurons. The OT was activated with a train of five stimuli at 10 Hz in the presence of muscarine alone (2 µM) and with sequential addition of blockers of L-type calcium channels (10 µM nimodipine) and NMDA receptors (5 µM R-CPP). Responses were measured in dLGN interneurons in current clamp (A, B, D, and E). Representative experiments in (A and B) and (D and E) show consecutive traces (gray) and their corresponding averages (black). Arrowheads indicate timing of stimulus train. (C) Summary of experiments as in (A and B) (n = 6). (F) Summary of experiments as in (D and E) (n = 4). Time scale bar in (A) applies to (A, B, D, and E). (0.80 MB TIF) [file pbio.1000348.s007.tif]
